# Future perspectives in melanoma research: Meeting report from the “Melanoma Bridge”. Napoli, December 1st–4th 2015

**DOI:** 10.1186/s12967-016-1070-y

**Published:** 2016-11-15

**Authors:** Paolo A. Ascierto, Sanjiv Agarwala, Gerardo Botti, Alessandra Cesano, Gennaro Ciliberto, Michael A. Davies, Sandra Demaria, Reinhard Dummer, Alexander M. Eggermont, Soldano Ferrone, Yang Xin Fu, Thomas F. Gajewski, Claus Garbe, Veronica Huber, Samir Khleif, Michael Krauthammer, Roger S. Lo, Giuseppe Masucci, Giuseppe Palmieri, Michael Postow, Igor Puzanov, Ann Silk, Stefani Spranger, David F. Stroncek, Ahmad Tarhini, Janis M. Taube, Alessandro Testori, Ena Wang, Jennifer A. Wargo, Cassian Yee, Hassane Zarour, Laurence Zitvogel, Bernard A. Fox, Nicola Mozzillo, Francesco M. Marincola, Magdalena Thurin

**Affiliations:** 1 IRCCS Istituto Nazionale Tumori, Fondazione “G. Pascale”, Naples, Italy; 2 Department of Oncology and Hematology, St. Luke’s University Hospital and Temple University, Bethlehem, PA USA; 3 Nanostring Inc., 500 Fairview Avenue N, Seattle, WA 98109 USA; 4 Division of Cancer Medicine, Department of Melanoma Medical Oncology, The University of Texas MD Anderson Cancer Center, Houston, TX USA; 5 Departments of Radiation Oncology and Pathology, Weill Cornell Medical College, New York, NY USA; 6 Skin Cancer Unit, Department of Dermatology, University Hospital Zürich, 8091 Zurich, Switzerland; 7 Gustave Roussy Cancer Campus Grand Paris, Villejuif, France; 8 Department of Surgery, Massachusetts General Hospital, Harvard Medical School, Boston, MA USA; 9 Department of Pathology, UT Southwestern Medical Center, Dallas, TX USA; 10 Departments of Medicine and of Pathology, Immunology and Cancer Program, The University of Chicago Medicine, Chicago, IL USA; 11 Department of Dermatology, Center for Dermato Oncology, University of Tübingen, Tübingen, Germany; 12 Fondazione IRCCS Istituto Nazionale dei Tumori, Milan, Italy; 13 Georgia Regents University Cancer Center, Georgia Regents University, Augusta, GA USA; 14 Yale University School of Medicine, New Haven, CT USA; 15 Departments of Medicine and Molecular and Medical Pharmacology, David Geffen School of Medicine and Jonsson Comprehensive Cancer Center at the University of California Los Angeles (UCLA), Los Angeles, CA USA; 16 Department of Oncology-Pathology, Karolinska Institute, Stockholm, Sweden; 17 Unit of Cancer Genetics, Institute of Biomolecular Chemistry, National Research Council, Sassari, Italy; 18 Department of Medicine, Memorial Sloan Kettering Cancer Center and Weill Cornell Medical College, New York, NY USA; 19 Department of Medicine, Early Phase Clinical Trials Program, Roswell Park Cancer Institute, New York, NY USA; 20 University of Michigan Comprehensive Cancer Center, Ann Arbor, MI USA; 21 University of Chicago, Chicago, IL USA; 22 Cell Processing Section, Department of Transfusion Medicine, Clinical Center, NIH, Bethesda, MD USA; 23 Departments of Medicine, Immunology and Dermatology, University of Pittsburgh, Pittsburgh, PA USA; 24 Department of Dermatology, Johns Hopkins University SOM, Baltimore, MD USA; 25 Istituto Europeo di Oncologia, Milan, Italy; 26 Division of Translational Medicine, Sidra Medical and Research Center, Doha, Qatar; 27 Genomic Medicine and Surgical Oncology, The University of Texas MD Anderson Cancer Center, Houston, TX USA; 28 The University of Texas MD Anderson Cancer Center, Houston, TX USA; 29 Gustave Roussy Cancer Center, U1015 INSERM, Villejuif, France; 30 University Paris XI, Kremlin Bicêtre, France; 31 Robert W. Franz Cancer Research Center, Earle A. Chiles Research Institute, Providence Cancer Center, Providence Portland Medical Center, Portland, OR USA; 32 Department of Molecular Microbiology and Immunology, Oregon Health and Science University, Portland, OR USA; 33 Cancer Diagnosis Program, National Cancer Institute, NIH, Bethesda, MD USA; 34 Unit of Medical Oncology and Innovative Therapy, Istituto Nazionale per lo Studio e la Cura dei Tumori “Fondazione G. Pascale”, Via Mariano Semmola, 80131 Naples, Italy

## Abstract

The sixth “Melanoma Bridge Meeting” took place in Naples, Italy, December 1st–4th, 2015. The four sessions at this meeting were focused on: (1) molecular and immune advances; (2) combination therapies; (3) news in immunotherapy; and 4) tumor microenvironment and biomarkers. Recent advances in tumor biology and immunology has led to the development of new targeted and immunotherapeutic agents that prolong progression-free survival (PFS) and overall survival (OS) of cancer patients. Immunotherapies in particular have emerged as highly successful approaches to treat patients with cancer including melanoma, non-small cell lung cancer (NSCLC), renal cell carcinoma (RCC), bladder cancer, and Hodgkin’s disease. Specifically, many clinical successes have been using checkpoint receptor blockade, including T cell inhibitory receptors such as cytotoxic T-lymphocyte-associated antigen 4 (CTLA-4) and the programmed cell death-1 (PD-1) and its ligand PD-L1. Despite demonstrated successes, responses to immunotherapy interventions occur only in a minority of patients. Attempts are being made to improve responses to immunotherapy by developing biomarkers. Optimizing biomarkers for immunotherapy could help properly select patients for treatment and help to monitor response, progression and resistance that are critical challenges for the immuno-oncology (IO) field. Importantly, biomarkers could help to design rational combination therapies. In addition, biomarkers may help to define mechanism of action of different agents, dose selection and to sequence drug combinations. However, biomarkers and assays development to guide cancer immunotherapy is highly challenging for several reasons: (i) multiplicity of immunotherapy agents with different mechanisms of action including immunotherapies that target activating and inhibitory T cell receptors (e.g., CTLA-4, PD-1, etc.); adoptive T cell therapies that include tissue infiltrating lymphocytes (TILs), chimeric antigen receptors (CARs), and T cell receptor (TCR) modified T cells; (ii) tumor heterogeneity including changes in antigenic profiles over time and location in individual patient; and (iii) a variety of immune-suppressive mechanisms in the tumor microenvironment (TME) including T regulatory cells (Treg), myeloid derived suppressor cells (MDSC) and immunosuppressive cytokines. In addition, complex interaction of tumor-immune system further increases the level of difficulties in the process of biomarkers development and their validation for clinical use. Recent clinical trial results have highlighted the potential for combination therapies that include immunomodulating agents such as anti-PD-1 and anti-CTLA-4. Agents targeting other immune inhibitory (e.g., Tim-3) or immune stimulating (e.g., CD137) receptors on T cells and other approaches such as adoptive cell transfer are tested for clinical efficacy in melanoma as well. These agents are also being tested in combination with targeted therapies to improve upon shorter-term responses thus far seen with targeted therapy. Various locoregional interventions that demonstrate promising results in treatment of advanced melanoma are also integrated with immunotherapy agents and the combinations with cytotoxic chemotherapy and inhibitors of angiogenesis are changing the evolving landscape of therapeutic options and are being evaluated to prevent or delay resistance and to further improve survival rates for melanoma patients’ population. This meeting’s specific focus was on advances in immunotherapy and combination therapy for melanoma. The importance of understanding of melanoma genomic background for development of novel therapies and biomarkers for clinical application to predict the treatment response was an integral part of the meeting. The overall emphasis on biomarkers supports novel concepts toward integrating biomarkers into personalized-medicine approach for treatment of patients with melanoma across the entire spectrum of disease stage. Translation of the knowledge gained from the biology of tumor microenvironment across different tumors represents a bridge to impact on prognosis and response to therapy in melanoma. We also discussed the requirements for pre-analytical and analytical as well as clinical validation process as applied to biomarkers for cancer immunotherapy. The concept of the fit-for-purpose marker validation has been introduced to address the challenges and strategies for analytical and clinical validation design for specific assays.

## Molecular and immune advances

The Cancer Genome Atlas (TCGA) identified four genetically defined subtypes of cutaneous melanoma: BRAF mutant, RAS mutant, NF1 mutant, and Triple Wild-Type. Mutations in each of the driver genes (BRAF, RAS, and NF1), contribute to deregulation of the mitogen activating protein kinase (MAPK/ERK) pathway, leading to uncontrolled cell growth. The most common subtype found was the BRAF subtype with 52% of cutaneous melanoma tumors harboring BRAF somatic mutations. Additional frequently affected molecular pathways identified through the TCGA analysis include the PI3K/AKT/mTOR (i.e., PTEN loss of function), cell cycle regulators (i.e., CdDKN2a, CDK4, CCND1), P53 (i.e., Tp53, MDM2), and epigenetic regulation (i.e., ARID2a) pathways [1].

PTEN is a negative regulator of PI3K in the PI3K/AKT/mTOR pathway. Complete loss of PTEN increases signaling through the PI3K-AKT signaling pathway, which is commonly assessed by measuring levels of phosphorylated (activated) AKT. Loss of function of PTEN is a frequent event in melanoma, particularly in tumors with BRAF(V600) mutations. Complete loss of PTEN expression correlates with shorter overall survival (OS) in patients with stage IIIB/C melanoma. Interestingly, loss of PTEN did not correlate with shorter time to distant metastasis, but instead specifically correlated with an increased risk of melanoma brain metastasis (MBM) [2]. In addition, analysis of tumors from patients that underwent resection of both brain and non-CNS metastases demonstrated that the MBMs were characterized by increased activation of the PI3K/AKT/mTOR pathway [3].

Gene expression profiling and synthetic lethality siRNAs screens in human melanoma cell lines implicated Oxidative Phosphorylation (OxPhos) in resistance to BRAF and MEK inhibitors [4]. The High OxPhos phenotype correlated with the expression of Peroxisome proliferator-activated receptor gamma coactivator 1-α (PGC1α), which is a transcriptional co-activator and a central inducer of mitochondrial biogenesis. Analysis of tumor biopsies from patients with acquired resistance to BRAF inhibitors demonstrated that ~50% of tumors had increased PGC1α expression compared to expression levels prior to treatment. Similarly, ~50% of human melanoma cell lines with de novo or acquired resistance to MAPK pathway inhibitors exhibited a High OxPhos phenotype. The High OxPhos cell lines were all sensitive to combined inhibition of mTORC1/2 and the MAPK pathway, whereas Low OxPhos cell lines were not. Focused studies demonstrated mTORC1/2 inhibition caused cytoplasmic sequestration of Microphthalmia-Associated Transcription Factor (MITF) and subsequent decreased expression of MITF-regulated genes, including PGC1-α. Experiments using human melanoma xenografts demonstrated that both mTORC1/2 inhibitors and a direct OxPhos inhibitor could inhibit the growth of High OxPhos, MAPKi-resistant melanomas in vivo.

Activation of the PI3K/AKT/mTOR pathway by loss of PTEN was also shown to promote resistance to T cell mediated cell killing in vitro and in vivo [5]. Loss of PTEN correlated with decreased CD8 T cell infiltrates in clinical specimens, as well as increased expression of several immunosuppressive cytokines. Further, melanoma patients with loss of PTEN expression had inferior clinical responses to anti-PD-1 therapy compared to patients with retained PTEN expression. While pan-PI3K inhibitors inhibited immune cell viability and function, a PI3K-β-isoform-selective inhibitor did not significantly affect immune function and induced synergy with checkpoint inhibitors in vivo.

These findings reinforce the significance of the PI3K/AKT/mTOR pathway in melanoma. OXPhos and mTOR are potential biomarkers to select patients for treatment with mTORi and the direct inhibitors of OXPhos as a new personalized therapeutic strategy. In addition, the findings support the rationale to combine PI3K/AKT/mTOR inhibitors with immunotherapy. Further investigations in this area, however, will need to balance anti-tumor effects, toxicities, and immune effects to fully realize clinical benefit, potentially through the use of isoform-selective inhibitors and/or novel dosing strategies.

The characterization of the mutational landscape of melanoma using the exome sequencing of 108 sun-exposed melanomas [6] at Yale University demonstrated the presence of genomic aberrations:( i) oncogenic mutations in RAC1, a GTPase member of the RAS superfamily, with RAC1^P295^ mutation affecting 4–7% of patients; (ii) recurrent mutations in PPP6C gene encoding serine/threonine-protein phosphatase 6 catalytic subunit regulating activity of the mitotic Aurora kinase A oncogene. At least 16 Aurora kinase inhibitors are in clinical studies, two of which (MLN8237/alisertib and GSK1070916A) are investigated in melanoma; and (iii) inactivating mutations in the neurofibromatosis type 1 (NF1) tumor suppressor gene known to negatively regulate RAS signaling. NF1 mutations occur in 30–45% of human melanomas that are BRAF/RAS WT, suggesting that NF1 may be a driver mutation in this subset, which, until now, was not amenable to targeted therapy approaches.

A recently carried out exome sequencing screen of 213 sun-exposed melanomas at Yale confirmed these findings and showed that NF1 is the third most frequently mutated driver gene in melanoma after BRAF and NRAS oncogenes [7]. In this cohort, inactivating NF1 mutations (mostly nonsense mutations, bi-allelic with loss of the WT allele) were found in 12% of patients, resulting in low NF1 protein expression and NRAS activation. Consequently, some NF1-mutant melanomas cell lines are sensitive to treatment with MEK inhibitors. NF1-mutant melanomas harbored concurrent MAPK pathway mutations, such as mutations in RASA2, including a recurrent mutation at position R511C observed in three NF1-mutant melanoma samples. The RASA2^R511C^ gene mutation that increases phospho-ERK (pERK) activation was recently found in a patient with Noonan syndrome, a known developmental syndrome defined as RASopathy. Rasopathies are caused by germline mutations in genes encoding transducers and modulator proteins participating in the RAS-MAPK signaling pathway including RASA2, PTPN11, SOS1, RAF1 (Noonan syndrome), protein tyrosine phosphatase, non-receptor type 11, PTPN11 (Leopard syndrome), and SPRED1 (Legius syndrome). There is clinical evidence for additive effect of NF1 and PTPN11 germline mutations that lead to severe/lethal forms of Noonan syndrome and neurofibromatosis [8]. Somatic mutations in PTPN11 have been also observed in several types of leukemia [9]. In total, 60% of melanomas with mutated NF1 also have mutations in PTPN11, SOS1, RASA2, SPRED1 and RAF1. The majority of gene mutations in NF1-mutant melanomas are the very same documented disease-causing mutations as seen in RASopathy patients.

The emerging mutational landscape in melanoma, which includes genes from several intracellular pathways, will enable patient targeted gene sequencing for determining melanoma diagnosis, prognosis and treatment options [10]. Targeted gene sequencing can distinguish benign from malignant melanocytic lesions, provides information regarding mutational evolution of in melanocytic lesion [11], assess drug sensitivity and resistance. The beneficial effects of BRAF inhibitors in melanoma patients bearing BRAF V600 mutations is well established, but the main issue remains the development of drug resistance, which is responsible for disease relapse within months after treatment. In most cases BRAFi resistant melanoma bear mutations reactivating MAPK pathway, e.g., MEK1 mutations and BRAF or KRAS amplification [12]. The observed frequent reactivation of MEK pathway in BRAFi resistant tumors led to the development of BRAFi + MEKi combination therapies, which improve survival but are unable to prevent disease relapse [13].

Progress in understanding of the evolution of resistance to targeted therapies in melanoma has been made in the recent years [14]. Two types of acquired BRAF inhibitor resistance have been proposed in this study: one caused by genetic variants such as double BRAF/NRAS mutant melanoma and another caused by a melanoma dramatically over-expressing a wild type receptor tyrosine kinase such as Platelet-derived growth factor receptor beta (PDGFRB). The consequences of these genetic versus non-genetic mechanisms are quite different. The genetic mechanism leads to reactivation or supra-baseline hyper-activation of the MAPK pathway and maintains MAPK-addiction. On the other hand, the non-genetic mechanism, while it still maintains tonically active MAPK signaling, has turned on alternative or MAPK-redundant growth and survival. Accordingly, addition of a MEK inhibitor would only re-sensitize the genetically driven resistant cells to the BRAF inhibitor since they’re still addicted to the MAPK pathway. MAPK-addicted can be distinguished from redundant resistance based on at several features. In MAPK-redundant resistance, phospho-ERK (pERK) levels and mutant BRAF signature output are still responsive to the BRAF inhibitor. Also, MAPK-redundant resistance is associated with transcriptome re-programming and a mesenchymal morphologic switch.

As more and more resistant cell lines and tumors from patients are analyzed, it became clear that, while genetic or sometime apparently non-genetic lesions (mutant BRAF alternative splicing which creates a N-terminally truncated BRAF) that hyper-activate the MAPK pathway may be different, they all render the resistant cells sensitive to further MAPK pathway suppression. From studies that compare patient-derived resistant tumors to their patient-matched baseline tumors, it has become clear that combination therapy with BRAF-MEK inhibitors has not exhausted the reservoir of rare genetic variants capable of supra-baseline MAPK hyper-activation [15]. Two examples of these unusual genetic configurations can be postulated. One is what we termed gene ultra-amplification selectively affecting mutant BRAF, resulting in back-to-back dimerization with CRAF. The other is concurrent BRAF and MEK mutations, resulting in a face-to-face interaction akin to the BRAF association with kinase suppressor of RAS (KSR) scaffolding protein. One consequence of a recalibrated MAPK signalsome is a striking phenotype of drug addiction where double-drug withdrawal led to slow-cycling (quiescent) or loss of viability.

Not all genetic lesions causing acquired resistance hit the MAPK pathway; a significant minority hit the PI3K-PTEN-AKT pathway. These genetic variants can in theory potentiate an adaptive response that occurs early on treatment and stem from both cancer cell-autonomous and paracrine mechanisms [16, 17]. However, genetic alterations did not seem adequate to explain the acquired resistant phenotype in patients [18]. Beyond mutant BRAF amplification and RAS (NRAS, KRAS) alterations, the other genetic mechanisms were not highly recurrent, and on the whole many cases of acquired resistance were unaccounted for by any specific genetic lesion. Deeper profiling and integrated analysis of the exome with the transcriptome and methylome across 48 pairwise before-and-after tumor comparisons demonstrated patterns of non-genomic and immune evolution of melanoma acquiring MAPKi resistance. Specifically, highly recurrent transcriptomic and correlated methylomic changes that supported alterations in a wide variety of cancer phenotypes were uncovered. Some of these changes (such as reduction in apoptotic sensitivity due to LEF1 and YAP1 alterations) are tumor cell-intrinsic, as supported by parallel functional analysis of MAPKi-resistant cell lines. Importantly, a significant fraction of MAPKi resistant tumors lose CD8 T infiltrating cells, suggesting loss of responsiveness to salvage anti-PD-1 therapy.

Thus, the stage is set to understand the origin of this omic-wide reprogramming, tumor heterogeneity, and co-evolution of the intra-tumoral immune microenvironment. To do so, it will be important to dissect alterations that take place early during MAPKi therapy, when the tumors are regressing and staying “dormant” or “quiescent” during the response period. From analysis of such tumors as well as cell lines and mouse models, the heterogeneous responses within tumor populations, their interactions, and temporal alterations before frank clinical relapse (within microscopic foci of resistance) can be appreciated. These insights might rationalize upfront combinations that truly restrict the bottleneck of melanoma evolution on MAPK targeted therapy. Thus, bioinformatics and system biology approaches are needed to integrate multi -omics databases to generate models for clinical melanoma management [19].

TCGA analysis also identified three clusters based on a transcriptomic classification of melanoma specimens. Discriminatory mRNA transcript profile enriched for immune gene expression associated with lymphocyte infiltrate based on pathology review and high lymphocyte specific protein tyrosine kinase (LCK) expression, was associated with improved patient survival and was named “immune” cluster [1]. A significant number of genes overexpressed in this subclass were associated with immune cell subsets (T cells, B cells, and NK cells), immune signaling molecules, co-stimulatory and co-inhibitory immune checkpoint proteins, cytokines, chemokines, and corresponding receptors. Importantly immune infiltration is statistically correlated with more favorable prognosis irrespective of genomic subtype designated by BRAF, RAS (N/H/K), NF1 mutations, and Triple-WT. The question of whether specific mutated melanoma antigens are responsible for differences in the degree of tumor infiltration by lymphocytes is an area of active investigation.

“Keratin” cluster represents biologically distinct melanoma subtype with adverse prognosis was characterized by high expression of genes associated with keratins, pigmentation, and epithelium, as well as genes associated with neuronal development or other organ-specific embryologic development and in addition included kallikreins and other epidermal genes. The ‘‘MITF-low’’ cluster was characterized by low expression of genes associated with pigmentation and epithelial expression including several MITF target genes and genes involved in immunomodulation, adhesion, migration, and extracellular matrix.

Also, global transcriptome also demonstrates immunomodulatory effects on melanoma in patients treated with sorafenib and dacarbazine. Upregulation of interferon (IFN)-stimulated immune response genes consistent with proinflammatory environment including IFNγ, T-cell infiltration and immune activation correlated with metabolic response assessed by PET-CT during sorafenib and dacarbazine therapy in patients with advanced melanoma. Induction of IFNγ stimulated genes correlating with increased level of serum IFNγ was found to be predictive of better clinical outcome [20].

Epigenetic regulation is also known to control tumor progression affecting a number of pathways (such as immune responsiveness, chemoresistance, stem-like behavior or apoptosis). Increased activity of the epigenetic modifier histone-lysine *N*-methyltransferase enzyme (EZH2) has been associated with different cancers and central role of EZH2 in promoting growth and metastasis of cutaneous melanoma have been found. EZH2 inactivation in melanoma-bearing mice stabilizes the disease through inhibition of growth and abolishment of metastases formation.

Comparably, in human melanoma cells, EZH2 inactivation impairs proliferation and invasiveness, accompanied by re-expression of tumour suppressor gene and increase patient survival. [21]. Whereas, increased expression of EZH2 in human melanoma was found to be associated with poor survival.

Adaptive mechanisms of drug resistance can also be linked to activation of receptor tyrosine kinases such as EGFR, IGF1R, PDGFR, AXL, EPHA2 [22]. Hyperactivation of ERBB3 receptor through phosporylation has been observed as an early feedback survival loop both in vitro and in vivo in response to RAF/MEK inhibition. This activation can be abrogated by anti-ERBB3 antibodies preventing the establishment of resistance to BRAF/MEK inhibitors in melanomas [23]. The feedback survival loop is promoted by increased autocrine production of EGFR ligand neuregulin, whose increased level of gene expression has been observed after BRAFi treatment in several cell lines and its secretion occurs shortly after melanoma cell exposure to BRAFi [24].

The combination of two monoclonal antibodies (mAbs) called A3 and A4 against two distinct epitopes of the extracellular domain of ERBB3 abrogates vemurafenib-induced ERBB3 activation, enhances inhibition of melanoma cells growth, and restores drug sensitivity to vemurafenib in BRAFi-resistant melanoma cells. It also reduced tumor relapse in an in vivo xenograft model when combined with vemurafenib and trametinib.

An alternative mechanism of resistance to targeted therapy involves miRNA expression. As miRNAs are master regulators of gene expression and miRNA deregulation impacts on several cellular processes such as cancer development, metastasis, invasion, migration and progression. Recently miRNA have emerged as a molecular regulator in the development and progression of melanoma. Study in melanoma cells led to identification of miR-579-3p targeting BRAF and MDM2 that controls growth and migration processes. Lower miR-579-3p level is observed in melanoma as compared to nevus, and higher levels correlate with good prognosis in melanoma patients. There are also accumulating data that miRNAs are involved in drug resistance and may be a biomarker to predict response to therapy. miR-579-3p was found to be downregulated in melanomas from patients who developed resistance to targeted therapies such as BRAFi and its level is inversely correlated with expression of target genes [25].

Investigating differentially expressed miRNAs in pre- and post-treatment melanoma biopsies may identify critical pathways hijacked in therapy-resistant tumors. Analysis of the miRNA expression profiles in BRAF-resistant tumors may enhance understanding of the biochemical mechanisms of resistance to targeted therapies.

CD8+ T cell-inflamed melanoma shows signs of increased immune suppressive mechanisms and anti-PD-1 therapy appears to be preferentially effective in T cell-inflamed tumors [26]. However, the underlying molecular mechanisms that can explain the absence of a T cell response in the majority of patients are not defined.

Exome sequencing and gene expression profiling of melanoma biopsies revealed activation of β-catenin in a 49% of non-T cell-infiltrated tumors. Using an inducible autochthonous mouse melanoma model (BRAF^V600E^/PTEN^−/−^ ± CAT-STA^+^; BP and BPC), a causal effect between tumor-intrinsic active β-catenin signaling and T cell exclusion was demonstrated [27]. Mechanistic studies revealed a lack of T cell priming against tumor-associated antigens in the context of β-catenin-expressing tumors. In-depth analysis indicated that absence of T cells was caused by defective recruitment of CD8α^+^ and CD103^+^ dermal dendritic cells (DC) into the tumor site, due to repressed expression of the chemokine CCL4. Furthermore, the knockdown of ATF3, a transcriptional repressor of CCL4, restores CCL4 expression in β-catenin^+^ tumor cells. Tumors expressing active β-catenin were resistant to therapy with anti-CTLA-4/anti-PD-L1 antibodies, mimicking the phenotype observed in humans [27]. The absence of CD103^+^ dendritic cells led to defective early T cell priming and absence of systemic immunity. However, whether tumor-intrinsic β-catenin signaling is responsible for mediating tumor resistance even after an anti-tumor T cell response is established remains elusive. To test this notion, the spontaneously rejected tumor cell line MC57.SIY, which is known to induce an immunologic memory mediating immune surveillance was used. Following rejection of MC57.SIY in BP-SIY and BPC-SIY hosts autochthonous tumors were induced. Although the primary SIY-specific CD8+ T cell response and the induced memory response were comparable between both tumor models, tumor protection was observed only against BP-SIY tumors, whereas it no protection BPC-SIY tumors. This increased tumor control in BP-SIY mice was accompanied by strong T cell infiltration and a boosted memory response. These results suggest that tumor-intrinsic β-catenin signaling might also be responsible for the observed exclusion of migrating effector T cells into the tumor site.

Taken together, these data provide strong evidence that up-regulation of β-catenin in tumor cells is a very potent mechanism of immune evasion against not only a primary immune response, but also against an immunologic memory. Moreover, tumor-intrinsic β-catenin activation likely mediates resistance not only to checkpoint blockade therapy but also to T cell adoptive transfer. Future studies will focus on therapeutic solutions targeting the activated β-catenin pathway with the intention to allow inflammation into this subset of tumors. In conclusion, tumor-intrinsic β-catenin signaling mediates lack of T cell infiltration and resistance towards checkpoint inhibition.

As one of the immune-based approach, adoptive cell therapy (ATC) has become an increasingly attractive modality for the treatment of patients with cancer. Endogenous population of tumor reactive T cell infiltrate is often absent in many solid tumor malignancies and transfer of such T cells to patients may lead to improved therapy. The augmentation of endogenous immune response may be obtained through autologous tumor-infiltrating lymphocytes (TILs), engineered T cells such as T cell receptor (TCR) modified T cells or chimeric antigen modified T cells (CAR), or circulating T cell therapy [28, 29]. Endogenous T cells (ETC) are derived from peripheral blood or tumor infiltrating lymphocytes as a source of effector cells for adoptive cell therapy.

Autologous effector enriched TILs were demonstrated as effective treatment for melanoma and potentially other tumors, although the antigen specificity in this infusion product has not been established. Disadvantages of TILs treatments include selection bias. TCR modified T cells and CARs provide antigen specificity by targeting specific peptide in the context of HLA or cell surface expressed antigen recognized by the antibody (Ab) recognition domain, respectively. Both TCR modified T cells and CAR products have shown efficacy in treatment of leukemia and other malignancies including melanoma. Disadvantages of using these cellular products are serious toxicities and safety/regulatory problems. ETC shows significant advantages having naturally occurring “self-selected” affinity. Moreover, peripheral blood as a source is very accessible and associated with low comorbidity. ETC is unfortunately time consuming, labor-intensive and technically challenging.

Rare population of tumor-reactive T cells present in the peripheral blood at frequencies as low as 1:100,000 or 0.001%, can be expanded in vitro up to >80% specific T cells in a population of 10^6^ cells. Preparation time can be significantly reduced by using clinical grade peptide-MHC-multimer-based sorting of antigen specific T cells. Strategies to enhance in vivo persistence of transferred T cells can lead to improved antitumor efficacy [28, 30–32]. However, the extrinsic (patient conditioning) and intrinsic factors (effector cells) contributing to long-term in vivo persistence are not well-defined. As a means to enhance persistence of infused T cells in vivo and to limit toxicity, lymphodepletion using cyclophosphamide alone can be administered as conditioning before infusing expanded peripheral blood mononuclear cell-derived, antigen-specific CD8+ cytotoxic T-lymphocyte (CTL) clones. In addition, IL-2 to build a better environment and priming with IL-21 in vivo generates CTL clones with prolonged in vivo survival. Adoptive T cell therapy using ETC represents feasible, effective and safe modality. ETC therapy can be applied to treat patients with melanoma and non-melanoma solid tumor malignancies. Modulation of intrinsic features of T cells that enhances in vivo persistence and extrinsic immunomodulation has potential to improve tumor immunorespose.

In vivo tracking revealed that the conditioning regimen provided a favorable milieu that enabled CTL proliferation early after transfer and localization to nonvascular compartments, such as skin and lymph nodes. CTL clones in the infusion product were characterized by an effector memory phenotype and CTL that persisted long term acquired phenotypic and/or functional qualities of central memory type CTLs in vivo [28, 33].

CTLA-4 is one of the checkpoint receptors expressed on T cells that provides inhibitory signals, establishing a negative feedback loop for T cell activation. The negative regulation of the immune response maintains peripheral tolerance to self-antigens and prevents damage to normal tissue. Blocking CTLA-4 may sustain the activation and proliferation of tumor-specific T cells, thus permitting the development of an effective tumor-specific immune response.

In a phase I/II trial of adoptive T cell therapy in combination with immune checkpoint blockade (anti-CTLA-4) in metastatic melanoma patients led to establishment of long-lived central memory T-cells. Evidence of epitope spreading was observed in patients with tumor regression/stable disease [28, 34].

## Combination therapies

Some tumors escape immune surveillance by upregulating a number of immunosuppressive pathways to inhibit the activity of tumor specific T cells. Tumor immunotherapy that targets T cells immunosuppressive mechanisms to unleash pre-existing anti-tumor immune response (e.g., immune checkpoints CTLA-4 and PD-1 or its receptor PD-L1) has shown success in some tumors [35]. But for the majority of tumors with limited numbers or no tumor infiltrating T cells additional interventions are needed to generate this immune response. Radiotherapy can be considered a good partner for immunotherapy because it is able not only to kill cancer cells but also to modify the tumor microenvironment, thus changing the immune system interaction with cancer potentially converting a lymphocyte-poor tumor in a lymphocyte-rich one [36].

In support of this concept, the combination of local radiotherapy (RT) with CTLA-4 blockade was proven to be effective in a mouse model of breast cancer refractory to anti-CTLA-4 alone [37] and to drive an oligoclonal expansion of CD8+ TILs [38]. Non-ablative, hypo-fractionated RT (6 Gy × 5 or 8 Gy × 3) regimens were shown to be effective in inducing anti-tumor immunity and abscopal effects, i.e., responses in non-irradiated tumors, in combination with anti-CTLA-4 [39]. The preclinical studies have been recently validated in a prospective phase II trial testing the combination of RT and ipilimumab in patients with metastatic NSCLC (NCT02221739), a disease poorly responsive to ipilimumab alone. Responses (CR + PR) were seen in 33% of the patients who completed treatment and 18% based on intent-to-treat [40].

Ongoing studies are aimed at improving RT-induced in situ vaccination. Generation of anti-tumor T cells at the irradiated tumor site is dependent on the balance of positive and negative signals that pre-exist or are induced by RT itself. Clearly, RT alone is seldom capable of inducing T cell-mediated rejection of aggressive poorly immunogenic tumors. One of the negative signals generated by RT is transforming growth factor (TGF)β. Activation of latent TGFβ by radiation-induced reactive oxygen species (ROS) hinders the activation of tumor-infiltrating dendritic cells (DC) and the priming of anti-tumor T cells. In this setting, TGFβ blockade showed a therapeutic synergy with radiation. Induced resistance in the tumor microenvironment with upregulation of PD-1 ligands limited responses, and PD-1 blockade was in fact able to extend survival of mice treated with the combination of radiation and TGFβ blockade and to delay tumor recurrence. Studies are ongoing to understand the mechanisms of tumor recurrence. Data suggests that TGFβ neutralization results in an increase in intratumoral Treg, and that another TGFβ family member, activin A, s responsible for this effect [41]. Combined TGFβ and activin A blockade reduced Treg and increased tumor-specific CD8 T cell responses, resulting in reduced recurrence rates. On the other hand, high dose radiotherapy leads to more release of ATP by dying cancer cells, which provides an activation signal to dendritic cells [42]. However, ATP can be rapidly converted to adenosine, which is immune-suppressive. This offers another potential target for intervention: blockade of adenosine generation improved recruitment and maturation of dendritic cells and tumor response to stereotactic body radiation therapy (SBRT) [43].

PI3K-AKT pathway inhibitors were found to selectively target T regulatory cell (Treg) CD25(+) FoxP3(+) with minimal effect on conventional T cells (Tconv). These results clearly show selective in vitro inhibition of activation (as represented by a decrease in downstream signaling) and proliferation of Treg in comparison to Tconv when treated with different AKT and PI3K inhibitors. This effect has been observed in both human and murine CD4 T cells [44]. Furthermore, it has been shown that PI3K-AKT inhibition enhances tumor antigen-specific vaccine efficacy and synergistically enhances anti-tumor responses [44]. In particular, AKT inhibition by MK-2206 enhances the anti-tumor therapeutic effect of tumor-specific vaccine; the PI3K inhibitor Wortmannin differentially affects proliferation of human Treg and Tconv cells. Similar results have been shown by inhibiting all class IA PI3K isoforms with the pan PI3K inhibitor GDC-0941. This observation suggests that Treg targeted therapies selectively reprogram Treg and represent an approach to circumvent a major element of immune suppression in patients with cancer.

A number of anti-tumor antibody-based therapies are aimed at direct killing of tumor cells or tumor microenvironment components including: (i) oncogenic receptors (Her2/neu, EGFR); (ii) non-oncogenic receptors (CD47), (iii) lineage specific molecules (CD20); (iv) tumor microenvironment (VEGFR, B7-H series); and (v) tumor specific antigens.

Anti-HER2/neu antibodies reduce tumor burden through blocking the HER2 oncogenic pathway and also by Fc receptor (FcR) mediated antibody-dependent cell-mediated cytotoxicity (ADCC). The therapeutic effect of anti-HER2/neu depends on natural killer (NK) cells, as depletion of NK cells significantly reduced efficacy of the treatment in mice. Antibodies have demonstrated an impact on immune response and tumor control in various xenografted tumor models. The involvement of effector T cells for Ab-mediated tumor regression is also essential for tumor control in immunocompetent and syngeneic host [45]. This process depends on type I and II IFN and Myeloid differentiation primary response gene 88 (MyD88) [46]. MYD88 gene encodes a cytosolic adapter protein that plays a central role in the innate and adaptive immune response. This protein functions as an essential signal transducer in the interleukin-1 and toll-like receptor signaling pathways and these pathways regulate that activation of numerous proinflammatory genes [47].

CD47 also known as integrin associated protein (IAP) is a transmembrane protein that in humans is encoded by the CD47 gene that is highly expressed on stem cells, including tumor stem cells. CD47 is involved in a range of cellular processes, including apoptosis, proliferation, adhesion, and migration. CD47 serves as the ligand for signal regulatory protein alpha (SIRPa), which is expressed on phagocytic cells including macrophages and dendritic cells, that when activated initiates a signal transduction cascade resulting in inhibition of phagocytosis. Considering that survival of tumor cells depends on the balance between “eat me” and “do not eat me” signaling, the question is whether anti-CD47 can be a candidate for targeting with therapeutic mAbs blocking a phagocytic inhibitory signal and inducing apoptosis. Anti CD47 antibodies were able to greatly reduce tumor burden and extend survival of human leukemia and in immunodeficient NSG mouse model [48].

The effect of anti-CD47 was also demonstrated to be based on CD8 T cells and its therapeutic effect depends on type I IFN responses [49]. However, neither MyD88 nor TIR-domain-containing adapter-inducing interferon-β (TRIF), that are two major downstream pathway members for IFN production are essential for anti-CD47-mediated tumor control. Thus, improved cytotoxic CD8+ T cells priming and increased DC cross-priming are the major mechanisms underlying the therapeutic effect of anti-CD47 Ab, the latter requiring type I IFN pathway. The fact that T cells demonstrate anti-tumor cytotoxic activity as a result of CD47-blocking antibody therapy could have important clinical implications. Anti-CD47 antibody—mediated phagocytosis of cancer by macrophages can initiate an antitumor T-cell immune response. Noteworthy, anti-CD47 antibody treatment not only enables macrophage phagocytosis of cancer, but also fosters the activation of tumor specific lymphocytes recognizing mutant proteins. Moreover, anti-CD47 Ab-mediated type I IFN induction by host DC that depends on activation of the stimulator of interferon genes (STING) pathway leads to adaptive immune responses against tumors. As a therapeutic approach, intratumoral injection of STING agonists has demonstrated profound therapeutic effects in multiple mouse tumor models, including melanoma, colon, breast, prostate, and fibrosarcoma. Better characterization of the STING pathway in human tumor recognition, and the development of new pharmacologic approaches to engage this pathway within the tumor microenvironment in patients, are important areas for clinical investigations. The experimental data suggests that anti-CD47 mAbs could be useful for monotherapy or as a component of combination treatment strategy for cancer treatment.

A critical checkpoint regulating the efficacy of T-cell-based cancer immunotherapy that correlate with tumor T cell infiltrate can be accumulation of multiple chemokine receptors on effector T cells and chemokine ligands within the tumor site [50]. Indeed, increased levels of chemokine receptors on T cells were shown to increase anti-tumor response to PD-1 therapy [51] whereas reduced chemokine expression by tumor cells increase resistance [27]. Chemokine targeting strategies further offer promise in combination with other immunotherapies that rescue CD8+ T cell function.

LIGHT [(homologous to Lymphotoxin (LT), inducible expression, competes with herpes simplex virus (HSV) glycoprotein D for HSV entry mediator (HVEM), a receptor expressed on T lymphocytes)] is a TNF family member that interacts with lymphotoxin receptor (LTR)3 and herpesvirus entry mediator (HVEM) expressed on stromal cells and T cells, respectively, and functions in T-cell responses. LIGHT systems also plays an important role in regulating expression of genes crucial for innate and adaptive defenses to pathogens and may contribute to immune tolerance in control of autoimmune diseases. LIGHT exhibits potent, CD28-independent costimulatory activity for T cell priming and expansion leading to enhanced T cell immunity against tumors by increasing tumor T cell infiltration and/or increased autoimmunity [52]. Targeting EGFR+ with anti-EGFR-LIGHT fusion protein in the tumor environment induces lymphotoxin beta receptor (LTβR)-associated chemokines and adhesion molecules that attract and prime naive T cells leading to the rejection of established, highly progressive tumors in mice [50]. It raises the possibility that tumor can be targeted with LIGHT to generate more CTL to convert TME into inflamed phenotype.

It is generally assumed that molecules have to be expressed on plasma cell membrane in order to be recognized by the corresponding antibodies in viable cells. This assumption has excluded the use of intracellular molecules (such as chaperons, transcription factors, signaling transduction components) as potential targets of antibody-based immunotherapy for the treatment of malignant diseases. This dogma has been challenged, since there is growing evidence that intracellular molecules may migrate to the plasma cell membrane in malignant cells. By panning a human phage display antibody library with a cultured human melanoma cell line we have isolated the single chain Fv(scFv) W9 which recognizes an extracellular epitope of the intracellular chaperone glucose-regulated protein of 94,000 daltons which is a member of the heat shock protein 90 family (Grp94). This molecule, which is a member of the heat shock protein 90 family plays an important role in the biology of malignant cells because of its asssociation with components of signaling pathways involved in cell proliferation, survival and migration [53]. The mAb W9 defined Grp94 epitope is expressed on various types of malignant cells including melanoma. In different types of cancer, the epitope is expressed not only on differentiated cancer cells, but also on cancer initiating cells (CICs). The mAb W9 defined Grp94 epitope is upregulated on BRAF^V600E^ melanoma cells upon treatment with BRAF inhibitors, and with chemotherapeutic agents. Furthermore the mAb W9 defined Grp94 epitope has a restricted distribution in normal tissues.

Therefore, Grp94 represents an attractive target for antibody-based immunotherapy of various types of solid tumors. The in vitro and in vivo anti-tumor activity of mAb W9 was investigated in a malignant melanoma model. The results indicate that mAb W9 inhibits the growth of melanoma cells. The antibody induces apoptosis as well as inhibits several signaling pathways (e.g., ERK, AKT and FAK). The anti-tumor activity of mAb W9 is enhanced by the small molecule LDE225, an inhibitor of the sonic hedgehog homolog (SHH) pathway. Furthermore, mAb W9 delays the development of BRAF inhibitor resistance in melanoma cells with mutant BRAF [54]. Lastly, mAb W9 induces the regression of experimental lung metastasis established in immunodeficient mice by intravenous injection of melanoma M21 cells. These data suggest that intracellular tumor antigens may be a useful source of targets for antibody-based immunotherapy of melanoma and other types of solid tumor and could be part of combination strategies with immunotherapeutic agents [55].

Intralesional therapy is a promising approach in melanoma treatment; especially for cutaneous metastases of metastatic melanoma that constitute a major clinical problem and that are accessible to injection in a high percentage of patients. The aim of this strategy is not only local control of melanoma, but potentially developing a systemic effect by stimulating an immune response after tumor injection.

A variety of oncolytic viruses are being tested in clinical trials including adenovirus, vaccinia, herpes, reovirus, Seneca Valley virus and coxsackievirus [56]. Locoregional therapies include oncolytic viruses such as Talimogene laherparepvec, Newcastle disease virus (NDV), Coxsackie virus A21, HF10-oncolytic HSV1 and Reovirus, and non-viral based therapies (PV-10/Rose Bengal disodium) demonstrated efficacy in patients with tumors with specific mutations or wild type tumors. Many questions, such as whether the combination of oncolytic viruses will provide synergy with other immunotherapy interventions and whether synergy might be a drug specific or specific for class of immunotherapy interventions remains to be tested. Considering that immune-active microenvironment and type I IFN transcriptional signature are associated with clinical benefit from immunotherapies, strategies targeting type I IFN pathway may sufficiently sensitize tumors to immune checkpoint blockade.

NDV has a potential as anti-cancer agent because it readily infects the majority of cancer cells because of ubiquitous expression of the viral receptor (containing sialic acid) and it is strong inducer of type I interferon and dendritic cells maturation. Clinical trials with systemically-administered NDV in humans demonstrated safety and durable clinical benefit in different cancer types. The durability of the clinical benefit suggested the strong immune component responsible for the treatment efficacy. Furthermore, NDV infection in animal model upregulated MHC and co-stimulatory molecules on the surface of tumor cells and delayed distant B16-F10 tumor growth, although only few complete regressions were observed. On the contrary, combination therapy of NDV transfection and CTLA-4 blockade lead to rejection of the locally administered with the virus and distant B16-F10 tumors and long-term survival in mice. In addition, the treatment induced inflammatory responses in distant tumors. Anti-tumor response of NDV and CTLA-4 blockage combination therapy is dependent on CD8 T cells, NK cells, and type I and II interferons [57].

Another unanswered question is whether there is a role for oncolytic virus therapies in patients who fail checkpoint inhibitors treatment. Coxsackievirus A21 (CVA21) is a naturally occurring Picornavirus, which CVA21 displays potent oncolytic activity in both in vitro cancer cells cultures and in vivo xenografts in mouse models of human cancers which exhibit high level of surface ICAM-1 expression. CVA21 rapidly replicates, rupturing the cells to release progeny virus particles and tumour antigens. Progeny virus continues the cycle by infecting and lysis of new cells, whereas tumor antigens activate the immune response [58]. CAV21 has shown an overall response rate of 26% and a disease control rate of 37% [59]. A phase 2 clinical study in humans demonstrated immune responses in injected lesions, non-injected visceral lesions and in distant non-injected visceral lesions. This strategy is tested as a rescue strategy to reconstitute the immune response within the tumor microenvinment (TME) in lesions resistant to immune checkpoint blockade [60].

The main intralesional agent currently in phase III trials is Talimogene laherparepvec (T-VEC) that is a HSV-1-derived oncolytic virus that has both a local effect (tumor cell lysis) and, to a lesser degree, a systemic effect (tumor-specific immune response) [61–68] T-VEC kills injected and non-injected A20 tumors in mice and induces protection against re-challenge [65]. In clinical trials, it has shown good safety profile and a durable response rate is (16%). For now, the use of T-VEC monotherapy appears to be limited to unresectable Stage III patients who are poor candidates for other therapies, or are refractory to other available therapies. In a phase 3 melanoma clinical study, T-VEC monotherapy demonstrated a significantly higher durable response rate (DRR, ≥6 mos response) versus GM-CSF. Current and upcoming trials will focus on finding the right virus and/or the right combination in the right population.

Combining T-VEC that promotes release of tumor-derived antigens with an immune checkpoint inhibitor that improves T cell responses such as ipilimumab significantly enhances efficacy of the combination as compared to either therapy alone. The phase 1b portion of a phase 1b/2 combination study (NCT01740297) completed enrollment and met its primary objective with no dose limiting toxicities and objective response rate (ORR) of 56% [69]. Also the combination of another oncolytic virus Pelareorep with anti-PD-1 MAb resulted in prolonged survival of mice injected with melanoma [70]. PV-10 is an investigational new drug containing a proprietary injectable formulation of Rose Bengal disodium (10% RB), a water-soluble xanthene dye currently in use in a topical ophthalmic diagnostic. PV-10 is designed for intralesional administration into solid tumors with an established safety history and prolonged retention in tumors. Preclinical data of combination of intralesional injection of PV-10 with anti-PD-1 are also promising [71]. PV-10 is able to selectively accumulate in lysosomes of cancer cells triggering the acute cytolysis mediated by lysosomes. Phase II results with this agent have shown promising results with both local and systemic effect [72]. Interesting results were also obtained in phase II trials with intratumoral electroporation (EP) of the plasmid containing IL-12 gene (IT-pIL12-EP) as well as coxsackievirus A21 (CVA21). The transfection of plasmid DNA-encoded IL-12 using leads to IL-12 expression at tumor site. IL-12 initiates local pro-inflammatory processes and a systemic anti-tumor immune responses. Patients with advanced melanoma, treated with pIL-12 monotherapy have benefited from a complete (14%) or partial response (17%), while the disease remained stable in 17% of patients and 52% of patients had a progressive disease (52%).

In conclusion, intralesional approaches may be applicable in melanoma treatment thanks to their local activity and the ability to trigger a systemic immune effect. Combination of locoregional therapy using oncolytic viruses with systemic immunotherapy such as checkpoint therapy appears to be safe and multiple agents and combinations are already in clinic or are in pre- and clinical development. Also, expanding the definition of “injectable lesion” to liver, deep lymph nodes and other locations would increase the efficacy of such therapies. However, further clinical studies and biomarkers are needed for optimal selection of patients for treatment with these agents to combine with other immunotherapy interventions and to improve the outcome of such strategies.

Immune checkpoint blockade with agents such as ipilimumab, pembrolizumab, and nivolumab has dramatically changed the outlook for patients with metastatic melanoma. Several studies have investigated the combination of various checkpoint blocking antibodies such as the combination of ipilimumab and nivolumab and the combination of ipilimumab and pembrolizumab. Questions remain about how to best combine or sequence these agents.

In a phase 2 study testing the combination of nivolumab + ipilimumab versus ipilimumab as first-line treatment for patients with advanced melanoma, the combination of nivolumab and ipilimumab compared to ipilimumab alone demonstrated a higher objective response rate by RECIST 1.1 (Response rate: 61 versus 11%, respectively) and progression free survival [73]. In the phase 3 study testing nivolumab + ipilimumab versus nivolumab versus ipilimumab, the combination of nivolumab + ipilimumab had the highest numeric overall response rate and progression free survival. While these efficacy results seem to favor combination immunotherapy, one must consider the higher rate of side effects with combination immunotherapy (approximately 55% of grade 3/4 immune-related adverse events). Fortunately, most adverse events resolve with immunosuppressant medications, and discontinuing immunotherapy due to side effects does not appear to affect efficacy of immunotherapy as 67.5% of patients who discontinued the combination due to side effects developed a response [74, 75].

The overall survival benefits of the combination compared to single agent PD-1 are not yet known. Nonetheless, the long-term overall survival rates in the phase I study of the combination of nivolumab + ipilimumab are impressive with 68% of patients alive at 3-years. The most important question, however, is whether there will be an overall survival advantage of the combination upfront versus single agent PD-1 first-line with ipilimumab (or the combination) as second-line therapy. Overall survival data from the phase 3 checkmate 067 study will be critical and determining the rate of cross-over to other immune checkpoint blocking antibodies in patients initially treated with single agent PD-1 and ipilimumab will be critical in this analysis.

Ideally there would be a biomarker to select the combination for patients who are most likely to benefit. Unfortunately, no biomarker, including PD-L1, is yet ready to be used for selecting patients for combination immunotherapy versus single agent PD-1 or ipilimumab. This remains a highly active area of research.

Locoregional interventions can markedly improve overall survival in stage IV melanoma, can contribute to palliative care to improve quality of life or to decrease symptoms as well as it can be combined with other therapy approaches including immunotherapy or target therapy. Numerous technical advances in diagnostic methodologies e.g., intraoperative ultrasound imaging (IOUS), magnetic resonance imaging (MRI), positron emission tomography (PET) and PET-computed tomography (PET/CT) provide 3-D tools for better diagnosis, staging and monitoring of the treatment of cancer including surgery.

Despite the major advances offered by new systemic therapies, surgery of stage IV melanoma remains an important therapeutic tool that can be used to rapidly and safely resolve localized disease. The rational for surgical resection as first option in stage IV melanoma is based on several factors. Single lesions are best treated by surgery while studies have that shown complete resection is possible in 25% of stage IV patients (M1a through M1c inclusive) [76]. The surgical procedure has acceptable morbidity and mortality and is associated with favorable survival rates [77]. Several prognostic factors for surgery in metastatic melanoma have been identified. In particular, patients who have limited sites of metastatic disease, prolonged disease-free survival and a tumor-volume doubling time of >60 days may be amenable to surgical resection. It is also important to consider whether the lesion can be completely resected. Complete surgical excision of limited metastatic disease can result in prolonged progression-free survival (PFS) in carefully selected patients. Surgery for distant metastatic melanoma, however, is rarely curative since the majority of patients with distant metastases have widespread micrometastatic disease even if clinical and imaging criteria suggest limited spread. Large tumor masses are difficult to eradicate with systemic therapy alone and surgery in combination with novel immuno- and targeted therapies can potentially improve clinical outcomes and/or patients’ quality of life. Indeed, surgery should be offered to reduce the target of subsequent adjuvant medical treatment whenever possible. Thus, reduction of the tumor mass which can be obtained with surgery is important in combination with immunotherapy.

For example, overall survival for patients with distant metastases (M1a) recurrence treated with surgery and with or without systemic medical therapy (SMT) versus SMT alone was retrospectively compared in the first Multicenter Selective Lymphadenectomy Trial (MSLT-I). Although all patients who underwent surgery had favorable survival (versus SMT alone), those with M1a metastases did particularly well. These patients had a median survival of greater than 60 months with surgery with or without SMT versus 12.4 months with SMT alone. These outcomes are superior to those on many patients with stage III metastases [77].

Surgery may also have an important role in combination with immunotherapy. Indeed, the removal of lesions that may be resistant to treatment with ipilimumab may improve outcomes for some patients. Pathological evaluation of the excised tissue is important to assess the presence of immune-infiltrate. In several cases, analysis of the excised tissue has revealed the presence of a diffuse immune infiltration which correlated with the outcome of these patients [78]. Also, surgery might be used as adjuvant, in combination with targeted therapy agents such as vemurafenib [79]. Thus, reduction of the tumor mass which can be obtained with surgery is important in combination with immunotherapy.

Surgery is very useful in well selected patients with stage IV melanoma and is associated with good outcomes and, in particular, combined modality approaches are likely to be most successful. Overall patients’ selection is paramount in order to identify patients most likely to benefit from surgery. Furthermore, clinical and translational studies are required to determine optimal combinations and treatment algorithms, but it appears that surgery should continue to play a prominent role.

## News on immunotherapy

Adjuvant therapies can have a fundamental role in improving the survival of melanoma patients diagnosed at earlier operable stages but continue to be at a high-risk of death from melanoma relapse (AJCC stages IIB-III). Three major meta-analysis studies of all randomized controlled trials (RCTs) of IFNα have supported its impact on relapse free survival (RFS) and overall survival (OS) of these patients [80]. Therefore, IFNα has been validated as a reference treatment in RCTs investigating new therapeutic agents for the adjuvant treatment of the high-risk population [81]. Positive results in terms of RFS were also achieved with adjuvant PEG IFNα2b administration in stage III patients, where the modest RFS benefit seen was still significant at 7.6 years of median follow up [82].

The results of a preplanned interim analysis of the phase III adjuvant bevacizumab study in patients with high risk melanoma at (AVAST-M) trial were reported by Corrie et al. [83]. This trial tested adjuvant bevacizumab versus observation in stage II/III resected melanoma patients (N = 1343). At a median follow-up of 25 months, overall survival and distant metastasis-free survival were similar among treatment arms and an improvement in the disease free interval (DFI) was observed (HR: 0.83; 95% CI 0.70–0.98; p = 0.03). Longer follow-up is needed to better assess the modest DFI benefit seen and to evaluate the effect on the primary endpoint of overall survival at 5 years.

Neoadjuvant therapy has improved the outcome of patients with multiple different solid tumors, including head and neck, breast, bladder, esophageal, and rectal cancers [84–87]. Benefits include improvements in survival, surgical resectability, local control, and organ preservation. Other advantages of neoadjuvant therapy are the ability to evaluate the clinical and pathologic responses and the potential to identify immunologic and histologic correlates of tumor response. Access to tumor tissue before and after neoadjuvant therapy also may allow a better understanding of the antitumor mechanisms of action that may enable more selective application of therapeutic agents to those patients who are more likely to benefit.

Patients with locoregionally advanced but surgically operable melanoma continue to carry a high risk of relapse and death despite the best available standard management approaches. Neoadjuvant studies targeting this patient population tested chemotherapy with temozolomide and biochemotherapy (BCT), in which BCT demonstrated high tumor response rates but was eventually abandoned with the failure of BCT to deliver survival benefits in randomized trials of metastatic disease. Smaller neoadjuvant immunotherapy studies with IFNα and ipilimumab have yielded promising clinical activity and important mechanistic insights and biomarker findings. Newer targeted and immunotherapeutic agents and combinations currently are being translated into the neoadjuvant setting at an accelerated pace and carry significant clinical promise. In drug development, the neoadjuvant approach allows access to blood and tumor tissue before and after initiation of systemic therapy, which allows for the conduct of novel mechanistic and biomarker studies in the circulation and the tumor microenvironment. Such studies may guide drug development and allow for the discovery of predictive biomarkers selected on the basis of their capacity to classify patients according to the degree of benefit from treatment or the risk for significant toxicity.

Neoadjuvant ipilimumab was tested in locoregionally advanced melanoma to evaluate safety and to define markers of activity and toxicity in the blood and tumor of patients at baseline and early on-treatment times [88]. Patients were treated with ipilimumab (10 mg/kg intravenously every 3 weeks for two doses) that bracketed surgery. Tumor and blood samples were obtained at baseline and at the definitive surgery time. Thirty-five patients were enrolled; stages IIIB (3 patients; N2b), IIIC (32 patients; N2c, N3), and IV (two patients). The worst toxicities included grade 3 diarrhea/colitis (five patients; 14%), hepatitis (two patients; 6%), rash (one patient; 3%), and elevated lipase (three patients; 9%). The median follow-up was 19 months. Among 33 evaluable patients, the preoperative radiologic assessment by PET-CT scans at 6–8 weeks after the initiation of ipilimumab revealed that three patients (9%) had objective responses (two patients, complete response; one patient, partial response). Twenty-one patients (64%) had stable disease, and eight patients (24%) experienced disease progression identified with PET-CT. [^18^F]-fludeoxyglucose PET/CT parameters at baseline (T0) and at the first scan after two doses of ipilimumab (T1) were unable to predict the risk of recurrence after surgery (at the significance level of 0.05). The number of lesions at T1 showed a trend towards predicting a higher chance of disease recurrence (p = 0.06). The median RFS was 11 months (95% CI, 6.2–19.2 months). Biomarker and mechanistic data nested within this study have supported the immunotherapeutic predictive value of the proinflammatory tumor microenvironment as measured by mRNA expression and CD8 T cell density at the tumor invasive margin [88]. Other studies have supported a role for the TH17 pathway in mediating immune related colitis after treatment with ipilimumab [89]. Ongoing studies are testing the neoadjuvant therapeutic value ipilimumab in combination with IFNα (NCT01608594), pembrolizumab in combination with IFNα (NCT02339324) and the combination of ipilimumab-nivolumab as compared to nivolumab alone (NCT02736123).

Among other ongoing targeted neoadjuvant combination studies are trials with targeted therapy agents vemurafenib/cobimetinib (NCT02303951, NCT02036086) and dabrafenib/trametinib (NCT01972347, NCT02231775). However, resistance ultimately develops in the majority of patients with metastatic disease, and several resistance mechanisms have been well characterized. Future studies optimizing MAPK pathway inhibition (targeting ERK and CDK4/6 as monotherapy and in combinations) and combinations that target alternate pathways implicated in mediating resistance (e.g., phosphoinositide 3-kinase [PI3K] and AKT), may take advantage of the neoadjuvant approach. Combination studies with immunotherapy (including IFN, IL-2, anti-CTLA4, and anti–PD-1/PD-1 ligand [PD-L1]) that are underway in patients with metastatic disease also may be transitioned into the neoadjuvant setting, supported by the hypothesis that the high response rates seen with BRAF/MEK inhibitors can be transformed into high durable response rates with immunotherapy [90, 91].

Other approaches for neoadjuvant therapy are intralesional approaches described earlier that have been shown to be relatively safe and well tolerated, with evidence of local and potential bystander/distant antitumor clinical activity that appears to be most promising with T-VEC at this time. This approach may provide a neoadjuvant therapeutic platform that can be combined with other immune-activating agents, including cytokines and checkpoint inhibitors. Combination studies of T-VEC with anti-CTLA4 and anti–PD-1 antibodies are underway in metastatic disease, and at least one neoadjuvant study with T-VEC monotherapy is planned in resectable regionally advanced melanoma (NCT02211131).

In conclusion, neoadjuvant therapy has the potential to improve the outcomes—including survival, surgical resectability, local control, and organ preservation—of patients with locoregionally advanced melanoma. Other advantages are the ability to evaluate the clinical and pathologic responses and the potential to identify immunologic and histologic correlates of tumor response. Access to tumor tissue before and after neoadjuvant therapy may allow a better understanding of the antitumor mechanisms of action that may enable more selective application of therapeutic agents to those patients who are more likely to benefit. Neoadjuvant immunotherapy with HDI and ipilimumab have yielded several important findings, and multiple studies involving newer immunotherapeutic and targeted agents and combinations are underway. Neoadjuvant therapy in melanoma continues to be investigational and should only be pursued in the context of a clinical trial [92].

The anti-CTLA-4 ipilimumab is an example of long-term survival benefits with the use of immunooncology compounds. Anti-CTLA-4 has shown to improve survival in melanoma patients and also to improve the long-term survival rate [93, 94]. Indeed, a median OS of 11.4 months and a OS of 22% at 3 years were reported [94]. The EORTC18071 study is a phase III trial compares adjuvant ipilimumab versus placebo after complete resection of high-risk stage III melanoma [95]. Patients with a single metastases in a sentinel node (SN) with a diameter <1 mm were excluded from this trial because of their excellent prognosis and very low relapse rate as recommended by the Rotterdam Criteria for tumor load in the SN [96–98]. Ipilimumab treatment was shown to improve relapse-free survival (RFS) significantly, with a hazard ration (HR) 0.74 (p < 0.0001) and to increase RFS rates at 2 and 3 years by 8 and 12% respectively, compared to placebo [99]. Adjuvant ipilimumab treatment was approved in 2015 by the FDA. With ipilimumab adjuvant therapy a benefit was seen across all subgroups. Patients with positive SN and patients with ulcerated primary lesions derived the biggest benefit from adjuvant ipilimumab. Thus the beneficial effect of ipilimumab is broader than with adjuvant IFN therapy as ulceration of the primary lesion is the overriding predictive factor of outcome with no benefit for non-ulcerated melanoma. This has been observed in the EORTC 18952 and 18991 trials in both a mature and long term analyses [82, 95, 100–102], in individual patient data meta-analyses (IPDMA) of EORTC trials [101] and individual patient data meta-analyses (IPDMA) of all 15 adjuvant trials reported thus far [103]. The safety profile of adjuvant use of ipilimumab at 10 mg/Kg was generally consistent with that observed in advanced melanoma, although the incidence of some immune-related adverse events (irAEs) (e.g., endocrinopathies) was higher in this study. Most patients came off treatment with ipilimumab after 4–5 doses because of irAE and 5 patients died of drug related causes [99].

Anti PD-1 agent nivolumab has been shown to improve overall survival and progression-free survival, as compared with dacarbazine, in previously untreated patients who had metastatic melanoma and that was significant in patients without a BRAF mutation [104]. More recently it has been demonstrated that nivolumab is equally effective in BRAF wild type patients and BRAF mutant patients alike [105]. Treatment of patients with advanced melanoma with the anti-PD-1 antibodies pembrolizumab and nivolumab has been demonstrated to be superior than treatment with ipilimumab alone [74, 106]. Recently, the EORTC1325 study has been activated, comparing another anti-PD-1 agent pembrolizumab therapy for 1 year versus placebo after complete resection of high-risk stage III melanoma. The EORTC trial will reach full accrual Q2-3 in 2016. In the USA a trial comparing adjuvant pembrolizumab versus high dose IFN therapy will be conducted. Moreover, and adjuvant trial comparing ipilimumab versus nivolumab in stage IIIB/C/resected stage IV has recently reached full accrual [107]. The profile of anti-PD1 antibodies in terms of high response rates and low toxicity is ideal for adjuvant use and the outcome of the trials is eagerly awaited [108].

Multiple mechanisms of melanoma-induced immune escape contribute to the failure of the spontaneous or vaccine-induced immune T cell responses to promote tumor regression in humans. In particular, a number of inhibitory pathways play a critical role in impeding T cell responses to tumor antigens (TAs), including PD-1, T-cell immunoglobulin and mucin-domain containing-3 (Tim-3) receptor, B- and T-lymphocyte attenuator (BTLA) and T-cell immunoreceptor with Ig and ITIM domains (TIGIT). These inhibitory receptors (IRs) are expressed by TA-specific CD8+ T cells in the tumor microenvironment (TME) while their respective ligands are expressed by antigen-presenting cells (APCs) and tumor cells. In patients with advanced melanoma, TA-specific CD8+ T cells present in the periphery and at tumor site co-express multiple IRs [109]. Circulating TA-specific CD8+ T cells and CD8+ tumor-infiltrating lymphocytes (TILs) that co-express PD-1, BTLA, and Tim-3 exhibit different level of T cell dysfunction [110, 111]. However, dysfunctional/exhausted CD8+ TILs are not totally inert and can exhibit cytolytic functions but are likely kept in check by the inhibitory pathways in the TME that can be released upon immune checkpoint blockade. Also, IRs can also be upregulated by activated T cells such as functional vaccine-induced CD8+ T cells in patients with advanced melanoma. In particular, PD-1 and Tim-3 regulate the expansion and function of vaccine-induced TA-specific CD8+ T cells, supporting combinatorial therapies with cancer vaccines and immune checkpoint blockade to increase the clinical efficacy of cancer vaccines [112].

Among IRs, TIGIT appears to represent an interesting target for the next generation of immune checkpoint blockade for several reasons. First, it is highly expressed by the majority of CD8+ TILs together with PD-1. Second, the TIGIT ligands, CD155/PVR, and CD112, are highly expressed in the TME by melanoma cells and APCs. Third, it has been shown that dual PD-1/TIGIT blockade augments the expansion and function of human TA-specific CD8+ T cells in vitro and promotes tumor rejection in animal models [113]. Interestingly, CD8+ TILs in melanoma downregulate the costimulatory molecule CD226, which competes with TIGIT for binding to the same ligands CD155 and CD112. Therefore, in addition to the TIGIT-mediated T-cell intrinsic inhibitory effects, the downregulation of CD226 expression resulting in the imbalance of TIGIT/CD226 expression by CD8+ TILs, may contribute to decrease T cell responses to melanoma [109, 113]. These data support implementation of dual PD-1/TIGIT blockade in clinical trials to determine its capability to increase the clinical benefits of monotherapy with PD-1 inhibitors in patients with advanced melanoma.

Strategies aimed at changing the tumor microenvironment (TME) include direct injection into tumor, checkpoint inhibition and vaccine/adjuvants. Early phase 2 trial data presented at the 2015 ASCO demonstrated by multi-spectral analysis that Coxsackievirus A21 induces both immune cell infiltration and up-regulation of immune response genes in the micro-environment of melanoma lesions. Similarly, adoptive immunotherapy with PD-1-deficient CD4+ tumor-specific T cells has been shown to augment therapeutic efficacy of tumor-specific CD8 T cells [114], presumably through an increase in infiltration and destruction of tumors.

Alternative approaches to change the TME include cancer vaccines. Short-lived proteins (SLiPs) and defective ribosomal products (DRiPs) are at the center of the MHC class I antigen processing pathway, linking immunosurveillance of viruses and tumors to mechanisms of specialized translation and cellular compartmentalization. Since SLiPs and DRiPs, are thought to represent the majority of epitopes presented by tumor cells and enable the immune system to rapidly detect alterations in cellular gene expression with great sensitivity [115], they represent a compelling choice for cancer vaccines. Methods to generate vaccines that contain SLiPs and DRiPs from cancer cells by inhibiting the proteasome and lysosomal degradation of resulting autophagic vesicles has been established. These vaccines have provided striking anti-cancer activity in a variety of preclinical animal models [116, 117]. Further, preliminary data suggests that the intranodal vaccination DRibbles+ CDN (cGAMP, STING Ligand) significantly augments the intratumoral CD8: FoxP3 ratio.

Recently, this approach was applied in clinical trials for patients with NSCLC and prostate cancer. DPV-001 is an off-the-shelf allogeneic DRibble vaccine developed from two human cancer cell lines (cGMP; adenocarcinoma and mixed histology (squamous/adeno)). The vaccine is composed of DC-targeted microvesicles containing natural agonists for TLR 2, 3, 4, 7 and 9, 15 DAMPs and more than 170 proteins overexpressed by the average NSCLC. Preliminary data from a phase II trial of cyclophosphamide with DPV-001 alone or with GM-CSF or imiquimod for adjuvant treatment of definitively treated stage IIIa or IIIb NSCLC showed that DPV-001 induced and boosted broad anti-cancer immunity in every patient [118]. This immunity was assessed by detection of IgG antibody responses using 9000 protein spotted arrays. Since development of IgG antibodies requires antigen-specific CD4 T cell help IgG responses can serve as a surrogate of CD4 T cell immunity [119].

T cells engineered to express high affinity T cell receptors or chimeric antigen receptors (CAR) have been successful for treating cancer and hematological malignancies. CD19- and CD22-CAR T cell therapy is currently being used children with acute lymphocytic leukemia (ALL), disialoganglioside GD2-CAR T cell therapy to treat children with osteosarcoma and neuroblastoma and B cell maturation antigen (BCMA)-CAR T cells to treat adults with multiple myeloma. A recent clinical trial of CD19-CAR T cell therapy revealed a 70% clinical complete response rate among twenty pediatric B-Cell ALL patients. Grade 4 cytokine release syndrome occurred in 14% of patients but all toxicities were reversible and prolonged B-cell aplasia did not occur [120]. Although CD19-CAR T cells therapy has been successful, some products fail to expand in culture.

CAR T cells are manufactured from autologous peripheral blood mononuclear cell (PBMC) concentrates which are enriched for lymphocytes prior to initiating the T cell culture and gene transfer. Potential causes for T cell expansion failure included, poor quality T cells (due to prior chemotherapy, underlying disease, or biological variability) and poor quality of the apheresis product (due to inhibition by contaminating cells). A review of 43 CD19-and 11 GD2-CAR T cell manufacturing records at one academic center found that for CD19-CAR T cells the yield of transduced cells was highly variable and 4 out of 28 CD19-CAR T cells products failed to meet transduced T cell dose criteria (1 or 3 × 10^6^ transduced T cells/kg) [121]. Further investigation showed that large quantities of monocytes and granulocytes in PBMC concentrates were associated with poor CD19-CAR T cell yields. In addition, although the CD19- and GD2-CAR T cell manufacturing methods were similar, the yield of GD2-CAR T cells was much lower than the yield of CD19-CAR T cells and the proportion of monocytes in the PBMC concentrates used to manufacture GD2-CAR T cells was much greater that of PBMCs used to manufacture CD19-CAR T cells. More rigorous monocyte depletion of the PBMC concentrates improved both CD19- and GD2-CAR T cell yields especially those for GD2-CAR T cells.

These results suggest that some autologous PBMC concentrates collected from patients with ALL, sarcoma or neuroblastoma contain large quantities of monocytic and granulocytic myeloid derived suppressor cells that inhibit T cell expansion. Furthermore, aggressive depletion of PBMCs of monocytes and granulocytes improves T cell expansion.

## Tumor microenvironment and biomarkers

Biomarkers can help with clinical decisions making for example predictive biomarkers to select patients who have a high likelihood of response to immunotherapy drugs. Other categories of biomarkers can serve different role in immune oncology (IO) as: (i) biomarkers before diagnosis that can be used for risk assessment and screening; (ii) at diagnosis biomarkers can assist with staging, grading, and therapy selection; and (iii) biomarkers also can be used to select additional therapy or for rational design of combination therapies or (iv) monitor for recurrent disease [122].

Immuno-therapeutic drug development now requires novel biomarker approaches considering the increasing number of immunotherapy agents available for the treatment of advanced cancers and the percentage of patients not responding to immune checkpoint inhibitors. Drug-specific predictive biomarkers would improve, at baseline for patient selection, benefit/risk and ultimately effectiveness. Also, biomarkers guiding biology-based combinations as well as optimal dose-schedule are still an unmet need.

Sources of inter-patient variability in IO include host germline polymorphisms in immune-regulatory genes, somatic alterations in tumor cells and environmental factors. Due to the complexity of the immune response and tumor biology it is unlikely that a predictive biomarker based on a single analyte will be very informative. Integrated model measuring different parameters including host, cancer and microbiota would be needed to predict with high accuracy which patient will benefit from which approach, single agent or combination.

Molecular barcoding is a proprietary (Nanostring) technology platform based on a single molecule fluorescent barcoding that allows for direct, digital, multiplexed measurements of gene expression from low amount of RNA without need for amplification with high precision and sensitivity (<1 copy per cell). It also has been optimized for formalin fixed paraffin embedded (FFPE) tissues/biopsies. Measurements are performed by the nCounter analysis instrument using ad hoc software that provides automatic quality control (QC), normalization and different visualization tools. Application of this technology to immune-oncology has been already demonstrated as (PAM50) gene signature panel showed prognostic value in breast cancer and received clearance by FDA under 510(k) regulation. Pancancer immune profiling panel provides a multiplexed gene expression probes designed to quantitate770 genes which fall under 4 categories:

- 24 different immune cell types in PBMC or tissue
- immunologic functions
- tumor specific antigens such as CT antigens
- housekeeping genes to facilitate sample to sample normalization

Nanostring platform has been used to study the relationship between immune gene signatures and clinical response to PD-1 blockade with pembrolizumab in patients with advanced solid tumors [123, 124]. Importantly those signatures have shown to be tumor type independent and to have higher negative predictive value (NPV) than immunohistochemistry (IHC)-based PD-L1 measurements. Next generation of biomarkers will need to measure and integrate the complexity of host, tumor and environment which will likely require measurement of different molecular entities i.e., multi-omics measurement of DNA, RNA, proteins, simultaneously (“3 D biology”) in the same sample (maximizing the amount and type of information obtained per sample) and same units.

Two broad categories of tumor escape based on cellular and molecular characteristics of the tumor microenvironment have been recently suggested. One major subset shows a T cell-inflamed phenotype consisting of infiltrating T cells, a broad chemokine profile and a type I interferon signature indicative of innate immune activation. These tumors appear to resist immune attack through the dominant inhibitory effects of immune system-suppressive pathways. Most immunotherapy responders including those treated with anti-PD1 have this phenotype. The other major phenotype lacks this T cell-inflamed phenotype and appears to resist immune attack through immune system exclusion or ignorance. These two major phenotypes of tumor microenvironment may require distinct immunotherapeutic interventions for maximal therapeutic effect [125]. If checkpoint blockade is preferentially active in T cell-inflamed tumors, then what molecular mechanisms explain the non-T cell-inflamed tumor microenvironment? Three major hypotheses have been proposed: (1) somatic differences at the level of tumor cells (as distinct oncogene pathways activated in different patients or mutational landscape and antigenic repertoire), (2) germline genetic differences at the level of the host (polymorphisms in immune regulatory genes), or (3) environmental differences (as commensal microbiota or immunologic/pathogen exposure history of patients).

In order to evaluate the mutational landscape and antigenic repertoire, malignant melanoma samples from TCGA were segregated based on T cell-inflamed gene signature. Expression of differentiation antigens and cancer-germline antigens was found to be comparable in T cell signature-high versus-low patients. Similarly, the overall mutational load (non-synonymous mutations) is comparable in T cell signature-high versus -low samples. Also, T cell signature-high and -low patients have equal patterns of predicted HLA-A0201 binding peptides. On the other hand, a minimal representation of so-called “tetrapeptide” sequences were observed in T cell signature-high versus -low tumors.

The second unanswered questions is, if overall antigen density is just as high in non-T cell-inflamed tumors, what is molecular explanation for absence of immune infiltrate [126]? A model of how melanoma-intrinsic β-catenin activation prevents host anti-tumor immune response has been proposed. Molecular analysis of human metastatic melanoma samples revealed a correlation between activation of the WNT/β-catenin signaling pathway and absence of a T-cell gene expression signature. The mechanism by which tumor-intrinsic active β-catenin signaling results in T-cell exclusion and resistance to anti-PD-L1/anti-CTLA-4 monoclonal antibody therapy was identified. Specific oncogenic signals, therefore, can mediate cancer immune evasion and resistance to immunotherapies, pointing to new candidate targets for immune potentiation [27].

The importance of commensal bacteria in explaining the different T cell-inflamed versus non-inflamed tumor microenvironments is based on their ability to shape systemic immunity. Jackson (JAX) and Taconic (TAC) mice exhibit robust versus weak anti-tumor immune responses and accumulation of intratumoral T cells. The difference between TAC and JAX mice was transferrable by co-housing or fecal transplant. The administration of JAX feces led to significantly improved tumor control and increased frequency of circulating antigen-specific T cells. The reciprocal transfer had little effect, consistent with cohousing experiments. Finally, the abundance of *Bifidobacterium* spp. correlated with antigen-specific T cell responses. Oral administration of *Bifidobacterium* mix to tumor-bearing TAC recipients improved tumor-specific immunity and response to αPD-L1 mAb. It is possible to conclude that T cell-inflamed tumor microenvironment may serve as a predictive biomarker for response to immunotherapies and the lack of the T cell-inflamed tumor microenvironment phenotype does not appear to be due to lack of antigens [127].

Mechanisms of PD-L1 expression may be either innate or adaptive, and the relative contribution of each mechanism varies by tumor type and even within tumor types. In melanoma, the adaptive resistance mechanism predominates [128]. PD-L1 mediated adaptive immune resistance may be described with T-cells recognizing tumor antigens and, as they are activated, they express PD-1, and secrete IFN-gamma as a part of their cytotoxic anti-tumor response, leading to PD-L1 upregulation on tumor cells and associated immune cells. PD-L1 expression in this setting can thus be thought of as reflecting an ongoing immune reaction against tumor, and as such, its expression may be used to predict responses to anti-PD-1-PD-L1 therapies [129, 130]. In tumors with constitutive PD-L1 expression, the predictive value of PD-L1 may be improved by adding an additional parameter such as infiltrating CD8+ T-cells or an IFN-gamma gene signature.

In addition, to the different mechanisms underlying PD-L1 expression, some of the variation reported in the predictive value of PD-L1 as a biomarker may be attributable to the different immunohistochemical (IHC) assays used for detection. The Blueprint Project is currently underway to characterize potential differences between the marketed diagnostic assays using the 28–8, 22C3, SP263, and SP142 monoclonal antibodies. When these four antibodies were compared in a laboratory derived test (rather than the marketed diagnostic assays), strong correlations were seen in levels of PD-L1 detection in melanoma samples. Most of the observed variation was attributable to geographic heterogeneity between different tumor sections as opposed to antibody performance. Next steps will likely focus on the reconciliation of the different marketed diagnostic assays amongst each other and with the standardization of evolving laboratory derived tests.

Other single markers have been nominated as predictors of response to anti-PD-1/PD-L1 therapies, including CD8 density and mutational load. The TCGA dataset was used to explore the relationship between PD-L1, a cytotoxic gene signature (CYT), mutational load and survival in patients with metastatic melanoma. Increasing values for all three parameters were associated with improved survival. Notably, CYT and PD-L1 expression were highly interdependent, while mutational load was not directly related to the presence of an inflamed tumor phenotype. Future biomarker panels will undoubtedly incorporate multiple parameters assessed in surgical pathology specimens, and studies focused on the integration and prioritization of distinct parameters beyond PD-L1 expression are currently underway.

The impact of the advances of the last few years on patients is tremendous. However, responses are heterogeneous and are not always durable. There is a critical need to better understand who will benefit from therapy, and this may best be accomplished through a deep molecular and immune analysis in samples from patients on therapy. In order to gain insight into response and resistance to targeted therapy for melanoma, serial biopsies (for genomic analysis and immune profiling) were performed in patients on BRAF inhibitors, pre-treatment, on-treatment, and at progression time points. Multiple molecular mechanisms of resistance have been identified. Oncogenic mutations contribute to tumor escape via multiple mechanisms. This is certainly the case in melanoma, where over half of patients have oncogenic mutations in the BRAF gene. Mutations in this gene lead to constitutive signaling though the MAPK pathway, with several deleterious effects, including uncontrolled proliferation, resistance to apoptosis, increased angiogenesis, invasion and metastasis, as well as immune evasion. By blocking oncogenic BRAF it is possible to abrogate the effects, actually making tumors more immunogenic. Immune mechanisms of response and resistance to targeted therapy were also identified, demonstrating that treatment with targeted therapy leads to a more favorable tumor microenvironment with increased melanoma antigens and CD8+ T cells and decreased immunosuppressive cytokines and VEGF. There is also an increase in expression of the immunomodulatory molecule PD-L1, suggesting a possible immune mechanism of resistance to therapy. These favorable immune changes are not likely to be directly related to increased melanoma antigen expression, but to overall changes in the microenvironment [131]. Investigating the antigen specificity of the infiltrating T cells, it resulted that treatment with targeted therapy results in a more clonal T cell response. Also, when the percent of pre-existing clones was plotted against treatment response, two groups clearly emerged: a group of patients with a low percentage of pre-existing clones who had a poor response to therapy and a group of patients with a high percentage of pre-existing clones who had a good response to therapy [132].

The hypothesis that combining targeted therapy and immune checkpoint blockade would enhance responses to therapy was then tested in a murine model and synergy was observed with delayed tumor outgrowth and prolonged survival when mice were treated with BRAF targeted therapy and PD-1 blockade, compared to either therapy alone [133].

Building on this data, the efforts were focused on better understanding responses to immune checkpoint blockade. To do this, a deep tissue-based analysis in longitudinal tumor samples from patients on immune checkpoint blockade (this group initially received Ipilimumab and then went onto PD-1 blockade therapy at progression) was performed. Then a deep molecular and immune profiling of these tumors was carried out to investigate whether molecular and immune “signatures” exist in pre-treatment, and early on-treatment samples of patients receiving CTLA-4 and PD-1 blockade may be predictive of response. Results demonstrated that immune signatures in early on-treatment tumor biopsies on PD-1 blockade were highly predictive of response [134]. It is hence possible to conclude that insights into mechanisms of therapeutic resistance to therapy can be gained through a deep analysis of molecular and immune signatures in patients, and could lead to identification of better biomarkers and strategies to overcome therapeutic resistance.

The rationale of studying myeloid cells and tumor exosomes for assessing and targeting immunosuppression is based on the evidence that tumors, through the release of systemic factors into the blood stream, can influence bone marrow myelopoiesis and promote the release of altered myeloid cells that can then feed the cancer site with immunosuppressive effects. The major population involved in this process is represented by myeloid-derived suppressor cells (MDSC) expressing either granulocytic or monocytic markers in both mice and humans, and exerting a pleiotropic suppression on antitumor T cells and NK cells through complex pathways. MDSC infiltrate tumor site where they can differentiate into tumor associated macrophages (TAM2) and sustain chronic inflammation. The immunosuppressive activity of monocytes from melanoma patients was investigated and CD14+ monocytes were found to exert immunosuppressive activity increased by 96 kDa heat shock protein (HSPPC-96)/GM-CSF vaccine, thus permitting to identify a new subset of MDSC potentially expandable by the administration of GM-CSF—based vaccines in metastatic melanoma patients [135].

Several studies have shown that tumor exosomes deliver information not only between tumor cells but also to other cell types, including different immune cell components. Exosomes are endosomal-derived nanovesicles released by most cells types, including tumor cells, and principally involved in intercellular communication in disease. There is increasing evidence that these extracellular vesicles (EVs), when released by malignant cells, may contribute to cancer progression by influencing different immune cell types, likely blunting specific T cell immunity and skewing innate immune cells toward a pro-tumorigenic phenotype. Because of this function and the ability to deliver molecular signals modulating neo-angiogenesis and stroma remodeling, tumor exosomes are believed to play a role in tumor progression by establishing metastatic niche [136]. Exosomes are crucial mediators of autocrine and paracrine intercellular trafficking of proteins and genetic material and thus are gaining attention as diagnostic biomarkers and therapeutic tool in different diseases including cancer.

Exosomes might be also involved in the generation of MDSC and in the cell-independent miRNA biogenesis and in the promotion of tumorigenesis. However, the question whether there is any link between tumor exosomes and MDSC and whether exosomes have the ability to mediate immune suppression remains unanswered. Preliminary data demonstrating the existence of this cross-talk (V. Umansky, DKFZ, Heidelberg, unpublished) showed that systemic injection of melanoma exosomes leads to MDSC induction in several immune sites including the bone marrow [137]. These data demonstrate the important role of miRNA in the immune regulation and suggest that the transfer of miRNA in exosomes from tumor to the host cells in tumor microenvironment is responsible for the changes in immunosuppressive phenotype. Melanoma cells with silenced miRNA, released exosomes that no longer carry this genetic material and, most importantly, no longer induce MDSC differentiation of normal monocytes. The evidence also suggests the occurrence of this pathway in melanoma patients, including the expression of MDSC-specific miRNA in circulating monocytes, tumor lesions, and plasma. Together with other soluble factors, tumor extracellular vescicles could be responsible for the MDSC expansion and activation of their immunosuppressive functions in a wide range of tumors. The possibility to identify and isolate cells that are the target of tumor miRNA will be crucial for the identification and analysis of novel pathways in stromal cells that support tumor growth.

It is therefore possible that the melanoma lesions release larger vesicles that target CD14+ cells and are responsible for the increased frequency of MDSC. Also, as MDSC specific miRNAs are present at higher levels in plasma of melanoma patients, they can serve as a plasma surrogate of MDSC activity and a potential plasma biomarker of myeloid dysfunctions and as a target for immunomodulation in melanoma patients. These data open new potential routes to the use of MDSC-specific miRNAs as biomarker and therapeutic target in clinical cancer setting.

The availability of immune prognostic factors will help to optimize adjuvant therapies in stage III metastatic melanoma. More than 125 immune parameters were investigated by flow cytometry and on paired blood and tumor specimen in 39 stage III melanoma patients. Results demonstrated that T cell exhaustion markers correlate best with prognostic clinical parameters and there is a high correlation between immunophenotypic biomarkers in blood and tumor. High frequencies of CD45RA+ CD4+ and CD3− CD56— tumor-infiltrating lymphocytes appear to be independent prognostic factors of short progression-free survival (PFS). Also, regulatory Treg within tumor-infiltrating lymphocytes are indicative of dismal prognosis, specifically in BRAF mutated melanoma. High natural toxicity receptor NKG2D expression on CD8+ tumor-infiltrating lymphocytes, low level of Treg, and low PD-L1 expression on circulating T cells predicted prolonged overall survival in the multivariate Cox analysis model [138].

Chemokines are critical regulators of leukocyte trafficking and immune functions. The expression patterns of nine homing receptors (CCR/CXCR) in naive and memory CD4+ and CD8+ T lymphocytes in 57 patients with metastatic melanoma (MM) with various metastatic sites were retrospectively analyzed to evaluate whether T cell CCR/CXCR expression correlates with intratumoral accumulation, metastatic progression, and/or overall survival (OS). Expression of homing receptor on lymphocytes strongly correlated with metastatic dissemination. Polyfunctional inflammatory cells such as CD8+ CCR6+ TEM and CD4+ CLA+ CCR10+ TEM that are TH2 cells present in blood are associated with bad prognosis in stage IV metastatic melanoma. Whereas CD8+ CCR9+ TN and CD4+ CXCR3+ TEM cells in blood are associated with more favorable prognosis in stage IV metastatic melanoma. Finally, in mice, CCR9/CCL25 and CXCR3 play complementary roles in natural immunosurveillance of cancer [139]. These data suggest that the interface between tumor and the host including activating pathways and tumor induced immunosuppressive mechanisms have to be analyzed on individual basis. Preliminary results have shown that each tumor draining lymph node of MM demonstrated individual reactivity profile to 11 different biomarkers detected with specific mAb, thus setting the stage for precision immunotherapy.

The role of intestinal dysbiosis should also be considered in response to immunotherapy in melanoma. The antitumor effects of CTLA-4 blockade depends on distinct *Bacteroides* species of gut microbiota. Link between CTLA-4 blockade, intestinal damage, dysbiosis, anti-microbial immune responses and anti-cancer immunity and rejection have been demonstrated [140]. Enterotyping of metastatic melanoma patients might therefore help to optimize responses to ipilimumab therapy. A clustering algorithm based on genus composition is currently under evaluation [140].

In 2014 the Society for Immunotherapy of Cancer (SITC) convened an Immune Biomarkers Task Force to review the state of art of existing assays and technologies, identify challenges for further success and provide recommendations for developing biomarkers for immunotherapy. The charge to one of the Working Group was to address pre-analytical and analytical as well as clinical and regulatory aspects of the validation process as applied to biomarkers for cancer immunotherapy.

Biomarker assay validation process can be separated into several continuous steps; assessment of basic assay performance (analytical validation); characterization of the performance of the assay with regard to its intended use (clinical validation); and validation in clinical trials that ensures that the assay performs robustly according to predefined specifications (fit-for-purpose) and facilitates the establishment of definitive acceptance criteria for clinical use (validation of clinical utility). A typical analytical validation plan involves several steps in which the assay must be optimized for multiple parameters: (i) sample-related (pre-analytic parameters), (ii) assay-related (analytical parameters); and (iii) data-related (post-analytical parameters) [141].

An important step in biomarker validation is the evaluation of pre-analytical factors that may affect assay performance due to specimen-related variability. For immunotherapies, there may be a need to monitor ex vivo immune responses in phenotypical or functional assays, which require high-quality samples to ensure reliable analytic output. To ensure that optimal pre-analytic processing regimens are followed, standard operating procedures (SOPs) for controlling specific biomarker development steps are essential.

Preanalytical processing of blood and analyte stability can be affected by the sample collection process including anticoagulants used for blood draws, freezing/thawing, time between collection and testing, and storage conditions before processing. Tissue based biomarkers can be measured on freshly frozen (FF) tumor samples or formalin fixed paraffin embedded (FFPE) tissue. FFPE tissue blocks are often available as archival materials as part of bio-banked samples for conventional immunohistochemistry (IHC). IHC is a multi-step diagnostic process that requires standardized conditions for tissue collection, fixation and processing, preparation of the IHC slide, and interpretation of the staining results. IHC based assays remain an important test as companion diagnostics (CDx) to assess antigen expression on diagnostic or surgical specimens for selecting patients and predicting patient-response to specific targeted therapy (e.g., HER2 expression for Herceptin or mutated BRAF for BRAF-inhibitor treatment), and more recently PD-L1 as companion diagnostic for pembrolizumab treatment of NSCLC patients [142].

General guidelines, including analyte stability and laboratory quality control, for performing analysis of tissue-based molecular biomarkers have been published [143].

Next generation sequencing (NGS) tests for tumor mutation analysis, similar to other complex molecular diagnostic tests, should demonstrate adequate analytical and clinical performance [144].

It should follow SOPs that specifically address materials and procedures including patient’s sample type, method of DNA extraction, as well as technical metrics for DNA quantification and quality, which can negatively impact on sensitivity and reproducibility of the assay [145].

Analytical Validation involves confirming that the assay used for the biomarker measurement has established: (i) Accuracy, (ii) Precision, (iii) Analytical sensitivity, (iv) Analytical specificity, (v) Reportable range of test results for the test system, (vi) Reference intervals (normal values) with controls and calibrators, (vii) Harmonized analytical performance if the assay is to be performed in multiple laboratories, (viii) Establishment of appropriate quality control measures. Depending on the particular category an assay can require distinct type of validation. Definite quantitative assays make use of calibrators and a regression model to calculate absolute quantitative values for unknown samples. The reference standard must be well defined and should be a representative of the biomarker. This type of assay can be accurate and precise. In relative-quantitative assays, reference calibrators can be used; however, because standards are not fully representative of the biomarker, assay precision can be validated, while the accuracy of the assay can only be estimated. Validation and maintaining reproducibility of multiparametric assays is much more challenging considering the number of analytic variables associated with high content assays (NanoString, single cell networ profiling (SCNP), mutational load, TCR sequencing). The capacity of high throughput platforms, such as NanoString or flow based analysis SCNP, enable multi-dimensional analysis of the immune system. Instead of detecting a single or limited number of molecular targets, assays are able to detect tens to hundreds of distinct molecular features simultaneously [146].

The post-analytical phase of biomarker evaluations involves data interpretation of the assay results. Dichotomous variables are relatively straightforward to incorporate into calculations of data sensitivity and specificity. However, most variables in measurement of immune response are continuous, resulting in variability with respect to analytical performance criteria and clinical relevance of the assay, e.g., cut-off points for clinical decision making. Essentially, a cut-off for classifying a sample as positive or negative needs to be determined empirically by correlating results with clinical outcomes in a clinical trial exploring efficacy of a drug.

As high throughput methods became widely available there is a need for computational methodologies for interpretation of the complex data for biological and clinical implications. Algorithms to develop multimodal signatures integrating various types of molecular tumor data (i.e., genomics, protein expression, functional etc.) with TME factors that reflect the complex biomarker information require the development of multifactorial classifiers/algorithms.

The final stage in the development of a biomarker predictive of clinical outcome or response is the assessment of its clinical validity and utility through the application of the analytically validated assay within a clinical trial, with multiple design options depending on the intended use of the test and availability of specimens from previous clinical trials.

Clinical validity relates to the observation that the predictive assay reliably divides the patient population(s) of interest into distinct groups with different expected outcomes to a specific treatment. The criteria for validation are defined by the nature of the question that the biomarker is intended to address (i.e., fit-for-purpose). A predictive biomarker needs to demonstrate the association with a specific clinical endpoint (e.g., survival or tumor response) in pre-treatment samples from patients that have been treated or exposed to a uniform treatment intervention. For example, the programmed cell death-1 protein ligand (PD-L1) immunohistochemistry (IHC 22C3 pharmDx) test was approved as a CDx to pembrolizumab (anti-PD-1 mAb inhibitor from Merck) as a single agent in second-line non-small cell lung cancer (NSCLC) [142].

There are multiple steps for biomarker clinical validation that encompass important elements of clinical study design and data analysis, including statistical assessments that rely on samples collected from prospective clinical trials or from archived samples that are well annotated with relevant clinical information. Clinical validation i.e., assessment of the test’s correlation with clinical outcome and the amount of improvement in patient outcomes its adoption would entail. The clinical sensitivity and specificity of the assay must be demonstrated through robust receiver operating characteristics (ROC) curves that provide support for the cut points, established using appropriate statistical analysis to identify responders versus non-responders. Statistically, several approaches can be applied for clinical validation of an assay. Internal validation can be achieved by using a study population that reflects the target population in which the test will be used. The study population is divided into two independent groups of specimens. One of these groups is the “training set”, i.e., the set of samples used to identify and characterize the “biomarker” (if single analyte) or to build a mathematical model or algorithm (in case of multi-variate assays). The second sample group is “the validation set” that is used to test whether the external validity of the biomarker/model is maintained in a sample cohort—independent from the training set.

There are three basic phase III design options that are frequently considered for assessing the ability of a biomarker to identify a subgroup of patients who will benefit from (or will not benefit from, and therefore should be avoiding) a new therapy. These are classified broadly into three categories: (i) The enrichment design; (ii) The stratified design; and (iii) The strategy design.

A clinical trial to evaluate the clinical utility of an omics test should be conducted with the same rigor as a clinical trial to evaluate a new therapy. This includes development of a formal protocol clearly detailing pre-specified hypotheses, study methods, and a statistical analysis plan. In some instances, a candidate predictive test for an existing therapy can be evaluated efficiently by using a prospective-retrospective design, in which the test is applied to archived specimens from a completed trial and the results are compared with outcome data that have already been collected. The “retrospective” aspect of this design requires that the assay can in fact be performed reliably on stored specimens [147].

In conclusion, immunotherapies have emerged as the most promising class of drugs to treat patients with cancer with diverse tumor types, however many patients do not respond to these therapies. Therefore, determining which patients derive clinical benefit from immune checkpoint agents remains an important clinical question and efforts to identify predictive markers of response are ongoing. The analytical and clinical validation of predictive biomarkers require appropriate clinical studies in which the evaluation of the clinical utility of the biomarker is a pre-specified endpoint of the study. A variety of study designs have been proposed for this purpose. Although, the randomized biomarker stratified design provides the most rigorous assessment of biomarker clinical utility, other study designs might be acceptable depending on the clinical context.

## Abbreviations

AE: adverse event
AJCC: American Joint Committee on Cancer
APC: antigen-presenting cell
ASCO: American Society of Clinical Oncology
ATC: adoptive cell therapy
BCT: biochemotherapy
BRAFi: BRAF inhibitor
CAR: chimeric antigen receptor
CCL: chemokine ligand
CD: cluster of differentiation
CIC: cancer initiating cell
CNS: central nervous system
CTL: cytotoxic T lymphocyte
CTLA-4: cytotoxic T lymphocyte antigen 4
CYT: cytotoxic gene signature
DC: dendritic cell
DFI: disease free interval
ETC: endogenous T cell
FDA: Food and Drug Administration
FFPE: formalin fixed paraffin embedded
GM-CSF: granulocyte-macrophage colony-stimulating factor
HLA: human leucocyte antigens
IFN: interferon
IHC: immunohistochemistry
IO: immuno-oncology
MAPK: mitogen-activated protein kinase
MBM: melanoma brain metastasis
MDSC: myeloid-derived suppressor cell
miRNA: microRNA
MM: malignant melanoma
NF1: neurofibromatosis type 1
NGS: next generation sequencing
NK: natural killer
NSCLC: non-small-cell lung cancer
OS: overall survival
PBMC: peripheral blood mononuclear cell
PD-1: programmed cell death protein 1
PD-L1: programmed death ligand 1
PET-CT: positron emission tomography-computed tomography
PFS: progression-free survival
RCC: renal cell cancer
RCT: randomized controlled trial
RFS: relapse free survival
RT: radiotherapy
SBRT: stereotactic body radiation therapy
SCNP: single cell networ profiling
SIRPa: signal regulatory protein alpha
SITC: Society for Immunotherapy of Cancer
SMT: systemic medical treatment
STING: stimulator of interferon genes
TCGA: the cancer genome atlas
TCR: T cell receptor
TGF: transforming growth factor
TLR: toll-like receptor
TIL: tumor-infiltrating lymphocyte
TME: tumor microenvironment
TNF: tumor necrosis factor
T-reg: regulatory T cells
WT: wild type

## References

[1] Cancer Genome Atlas (2015). Genomic classification of cutaneous melanoma. Cell.

[2] Bucheit AD, Chen G, Siroy A (2014). Complete loss of PTEN protein expression correlates with shorter time to brain metastasis and survival in stage IIIB/C melanoma patients with BRAFV600 mutations. Clin Cancer Res.

[3] Chen G, Chakravarti N, Aardalen K (2014). Molecular profiling of patient-matched brain and extracranial melanoma metastases implicates the PI3K pathway as a therapeutic target. Clin Cancer Res.

[4] Gopal YN, Rizos H, Chen G (2014). Inhibition of mTORC1/2 overcomes resistance to MAPK pathway inhibitors mediated by PGC1alpha and oxidative phosphorylation in melanoma. Cancer Res.

[5] Peng W, Chen JQ, Liu C (2016). Loss of PTEN promotes resistance to T cell-mediated immunotherapy. Cancer Discov.

[6] Krauthammer M, Kong Y, Ha BH (2012). Exome sequencing identifies recurrent somatic RAC1 mutations in melanoma. Nat Genet.

[7] Krauthammer M, Kong Y, Bacchiocchi A (2015). Exome sequencing identifies recurrent mutations in NF1 and RASopathy genes in sun-exposed melanomas. Nat Genet.

[8] Thiel C, Wilken M, Zenker M (2009). Independent NF1 and PTPN11 mutations in a family with neurofibromatosis-Noonan syndrome. Am J Med Genet A.

[9] Kontaridis MI, Swanson KD, David FS (2006). PTPN11 (Shp2) mutations in LEOPARD syndrome have dominant negative, not activating, effects. J Biol Chem.

[10] Hovelson DH, McDaniel AS, Cani AK (2015). Development and validation of a scalable next-generation sequencing system for assessing relevant somatic variants in solid tumors. Neoplasia.

[11] Shain AH, Yeh I, Kovalyshyn I (2015). The Genetic evolution of melanoma from precursor lesions. N Engl J Med.

[12] Little AS, Smith PD, Cook SJ (2013). Mechanisms of acquired resistance to ERK1/2 pathway inhibitors. Oncogene.

[13] Robert C, Karaszewska B, Schachter J (2015). Improved overall survival in melanoma with combined dabrafenib and trametinib. N Engl J Med.

[14] Nazarian R, Shi H, Wang Q (2010). Melanomas acquire resistance to B-RAF(V600E) inhibition by RTK or N-RAS upregulation. Nature.

[15] Moriceau G, Hugo W, Hong A (2015). Tunable-combinatorial mechanisms of acquired resistance limit the efficacy of BRAF/MEK cotargeting but result in melanoma drug addiction. Cancer Cell.

[16] Shi H, Hong A, Kong X (2014). A novel AKT1 mutant amplifies an adaptive melanoma response to BRAF inhibition. Cancer Discov.

[17] Obenauf AC, Zou Y, Ji AL (2015). Therapy-induced tumour secretomes promote resistance and tumour progression. Nature.

[18] Hugo W, Shi H, Sun L (2015). Non-genomic and immune evolution of melanoma acquiring MAPKi resistance. Cell.

[19] Dummer R, Goldinger SM, Paulitschke V (2015). Curing advanced melanoma by 2025. Curr Opin Oncol.

[20] Urosevic-Maiwald M, Barysch MJ, Cheng PF (2015). Profiling reveals immunomodulatory effects of sorafenib and dacarbazine on melanoma. Oncoimmunology.

[21] Zingg D, Debbache J, Schaefer SM (2015). The epigenetic modifier EZH2 controls melanoma growth and metastasis through silencing of distinct tumour suppressors. Nat Commun.

[22] Holderfield M, Deuker MM, McCormick F (2014). Targeting RAF kinases for cancer therapy: BRAF-mutated melanoma and beyond. Nat Rev Cancer.

[23] Fattore L, Malpicci D, Marra E (2015). Combination of antibodies directed against different ErbB3 surface epitopes prevents the establishment of resistance to BRAF/MEK inhibitors in melanoma. Oncotarget.

[24] Fattore L, Marra E, Pisanu ME (2013). Activation of an early feedback survival loop involving phospho-ErbB3 is a general response of melanoma cells to RAF/MEK inhibition and is abrogated by anti-ErbB3 antibodies. J Transl Med.

[25] Fattore L, Acunzo M, Romano G, et al. miR-579-3p is a novel master regulator of melanoma progression and drug resistance metastatic melanoma. Proceedings: AACR 107th Annual Meeting 2016 April 16–20, 2016; New Orleans.

[26] Spranger S, Spaapen RM, Zha Y (2013). Up-regulation of PD-L1, IDO, and T(regs) in the melanoma tumor microenvironment is driven by CD8(+) T cells. Sci Transl Med.

[27] Spranger S, Bao R, Gajewski TF (2015). Melanoma-intrinsic beta-catenin signalling prevents anti-tumour immunity. Nature.

[28] Chapuis AG, Roberts IM, Thompson JA (2016). T-cell therapy using interleukin-21-primed cytotoxic T-cell lymphocytes combined with cytotoxic T-cell lymphocyte antigen-4 blockade results in long-term cell persistence and durable tumor regression. J Clin Oncol.

[29] Yee C (2014). The use of endogenous T cells for adoptive transfer. Immunol Rev.

[30] Chapuis AG, Thompson JA, Margolin KA (2012). Transferred melanoma-specific CD8+ T cells persist, mediate tumor regression, and acquire central memory phenotype. Proc Natl Acad Sci USA.

[31] Rosenberg SA, Dudley ME (2009). Adoptive cell therapy for the treatment of patients with metastatic melanoma. Curr Opin Immunol.

[32] Zhou J, Dudley ME, Rosenberg SA (2005). Persistence of multiple tumor-specific T-cell clones is associated with complete tumor regression in a melanoma patient receiving adoptive cell transfer therapy. J Immunother.

[33] Li Y, Bleakley M, Yee C (2005). IL-21 influences the frequency, phenotype, and affinity of the antigen-specific CD8 T cell response. J Immunol.

[34] Chapuis AG, Lee SM, Thompson JA (2016). Combined IL-21-primed polyclonal CTL plus CTLA4 blockade controls refractory metastatic melanoma in a patient. J Exp Med.

[35] Sharma P, Allison JP (2015). The future of immune checkpoint therapy. Science.

[36] Demaria S, Formenti SC (2012). Role of T lymphocytes in tumor response to radiotherapy. Front Oncol.

[37] Demaria S, Kawashima N, Yang AM (2005). Immune-mediated inhibition of metastases after treatment with local radiation and CTLA-4 blockade in a mouse model of breast cancer. Clin Cancer Res.

[38] Pilones K, Koelwyn G, Emerson R (2015). Unique changes in the TCR repertoire of tumor-infiltrating lymphocytes underlie the synergy of radiotherapy with CTLA-4 blockade. Cancer Res.

[39] Dewan MZ, Galloway AE, Kawashima N (2009). Fractionated but not single-dose radiotherapy induces an immune-mediated abscopal effect when combined with anti-CTLA-4 antibody. Clin Cancer Res.

[40] Golden EB, Chachoua A, Fenton-Kerimian MB (2015). Abscopal responses in metastatic non-small cell lung cancer (NSCLC) patients treatded on a phase 2 study of combined radiation therapy and ipilimumab: evidence for the in situ vaccination hypothesis of radiation. Int J Radiat Oncol Biol Phys.

[41] VanpouilleBox C, Formenti S, Demaria S (2015). TGFb and activin A control regulatory T cells in irradiated tumors. J Immunother Cancer..

[42] Golden EB, Frances D, Pellicciotta I (2014). Radiation fosters dose-dependent and chemotherapy-induced immunogenic cell death. Oncoimmunology.

[43] Wennerberg E, Kremer V, Childs R (2015). CXCL10-induced migration of adoptively transferred human natural killer cells toward solid tumors causes regression of tumor growth in vivo. Cancer Immunol Immunother.

[44] Abu-Eid R, Samara RN, Ozbun L (2014). Selective inhibition of regulatory T cells by targeting the PI3K-Akt pathway. Cancer Immunol Res.

[45] Park S, Jiang Z, Mortenson ED (2010). The therapeutic effect of anti-HER2/neu antibody depends on both innate and adaptive immunity. Cancer Cell.

[46] Stagg J, Loi S, Divisekera U, Ngiow SF, Duret H, Yagita H, Tenga MW, Smytha MJ (2011). Anti–ErbB-2 mAb therapy requires type I and II interferons and synergizes with anti-PD-1 or anti-CD137 mAb therapy. Proc Natl Acad Sci USA.

[47] Gajewski TF, Schreiber H, Fu Y-X (2013). Defective IFN production can reduce cross priming while targeting tumor tissues with type I IFN can bridge innate and adaptive immune responses. Nat Immunol.

[48] Chao MP, Majeti R, Weissman IL (2012). Programmed cell removal: a new obstacle in the road to developing cancer. Nat Rev Cancer.

[49] Liu X, Pu Y, Cron K (2015). CD47 blockade triggers T cell-mediated destruction of immunogenic tumors. Nat Med.

[50] Tang H, Wang Y, Chlewicki LK (2016). Facilitating T cell infiltration in tumor microenvironment overcomes resistance to PD-L1 blockade. Cancer Cell.

[51] Peng D, Kryczek I, Nagarsheth N (2015). Epigenetic silencing of TH1-type chemokines shapes tumour immunity and immunotherapy. Nature.

[52] Chen L (2004). Co-inhibitory molecules of the B7-CD28 family in the control of T-cell immunity. Nat Rev Immunol.

[53] Wu BX, Hong F, Zhang Y (2016). GRP94/gp96 in cancer: biology, structure, immunology, and drug development. Adv Cancer Res.

[54] Sabbatino F, Favoino E, Wang Y (2015). Grp94-specific monoclonal antibody to counteract BRAF inhibitor resistance in BRAFV600E melanoma. J Transl Med.

[55] Wang Y, Wang X, Ferrone CR (2015). Intracellular antigens as targets for antibody based immunotherapy of malignant diseases. Mol Oncol.

[56] Kaufman HL, Kohlhapp FJ, Zloza A (2015). Oncolytic viruses: a new class of immunotherapy drugs. Nat Rev Drug Discov.

[57] Zamarin D, Holmgaard RB, Subudhi SK (2014). Localized oncolytic virotherapy overcomes systemic tumor resistance to immune checkpoint blockade immunotherapy. Sci Transl Med.

[58] Andtbacka RHI (2013). 8th World Congress of melanoma.

[59] Andtbacka RHI, Curti B, Kaufman H (2014). Secondary endpoints of a Phase II study of a novel oncolytic immunotherapeutic agent, Coxsackievirus A21, delivered intratumorally in patients with advanced malignant melanoma.

[60] Andtbacka RH, Curti BD, Kaufman H (2015). Final data from CALM: A phase II study of Coxsackievirus A21 (CVA21) oncolytic virus immunotherapy in patients with advanced melanoma. J Clin Oncol.

[61] Varghese S, Rabkin SD (2002). Oncolytic herpes simplex virus vectors for cancer virotherapy. Cancer Gene Ther.

[62] Hawkins LK, Lemoine NR, Kirn D (2002). Oncolytic biotherapy: a novel therapeutic plafform. Lancet Oncol.

[63] Fukuhara H, Todo T (2007). Oncolytic herpes simplex virus type 1 and host immune responses. Curr Cancer Drug Targets.

[64] Sobol PT, Boudreau JE, Stephenson K (2011). Adaptive antiviral immunity is a determinant of the therapeutic success of oncolytic virotherapy. Mol Ther.

[65] Liu BL, Robinson M, Han ZQ (2003). ICP34.5 deleted herpes simplex virus with enhanced oncolytic, immune stimulating, and anti-tumour properties. Gene Ther.

[66] Melcher A, Parato K, Rooney CM (2011). Thunder and lightning: immunotherapy and oncolytic viruses collide. Mol Ther.

[67] Dranoff G (2003). GM-CSF-secreting melanoma vaccines. Oncogene.

[68] Andtbacka RH, Kaufman HL, Collichio F (2015). Talimogene laherparepvec improves durable response rate in patients with advanced melanoma. J Clin Oncol.

[69] Puzanov I, Milhem MM, Minor D (2016). Talimogene Laherparepvec in combination with ipilimumab in previously untreated, unresectable stage IIIB–IV melanoma. J Clin Oncol.

[70] Rajani K, Parrish C, Kottke T (2016). Combination therapy with reovirus and anti-PD-1 blockade controls tumor growth through innate and adaptive immune responses. Mol Ther.

[71] Pilon-Thomas S, Liu H, Kodumudi K. Efficacy of intralesional injection with PV-10 in combination with co-inhibitory blockade in a murine model of melanoma. Society for Immunotherapy of Cancer Annual Meeting; 2014.

[72] Agarwala SS (2015). Intralesional therapy for advanced melanoma: promise and limitation. Curr Opin Oncol.

[73] Postow MA, Chesney J, Pavlick AC (2015). Nivolumab and ipilimumab versus ipilimumab in untreated melanoma. N Engl J Med.

[74] Larkin J, Chiarion-Sileni V, Gonzalez R (2015). Combined nivolumab and ipilimumab or monotherapy in untreated melanoma. N Engl J Med.

[75] Larkin J, Hodi FS, Wolchok JD (2015). Combined nivolumab and ipilimumab or monotherapy in untreated melanoma. N Engl J Med.

[76] Hoshimoto S, Faries MB, Morton DL (2012). Assessment of prognostic circulating tumor cells in a phase III trial of adjuvant immunotherapy after complete resection of stage IV melanoma. Ann Surg.

[77] Howard JH, Thompson JF, Mozzillo N (2012). Metastasectomy for distant metastatic melanoma: analysis of data from the first multicenter selective lymphadenectomy trial (MSLT-I). Ann Surg Oncol.

[78] Simeone E, Gentilcore G, Giannarelli D (2014). Immunological and biological changes during ipilimumab treatment and their potential correlation with clinical response and survival in patients with advanced melanoma. Cancer Immunol Immunother.

[79] Amaravadi RK, Kim KB, Flaherty KT, et al. Prolonged responses to vemurafenib in patients with BRAFV600E mutant melanoma with low tumor burden at baseline. 8th International Congress of the Society for Melanoma Research; Tampa: 2013.

[80] Tarhini AA, Gogas H, Kirkwood JM (2012). IFN-alpha in the treatment of melanoma. J Immunol.

[81] Mocellin S, Lens MB, Pasquali S, Pilati P, Chiarion Sileni V. Interferon alpha for the adjuvant treatment of cutaneous melanoma. Cochrane Database Syst Rev. 2013(6):CD008955. 10.1002/14651858.CD008955.pub2 PMC10773707 23775773

[82] Eggermont AM, Suciu S, Testori A (2012). Long-term results of the randomized phase III trial EORTC 18991 of adjuvant therapy with pegylated interferon alfa-2b versus observation in resected stage III melanoma. J Clin Oncol.

[83] Corrie PG, Marshall A, Dunn JA (2014). Adjuvant bevacizumab in patients with melanoma at high risk of recurrence (AVAST-M): preplanned interim results from a multicentre, open-label, randomised controlled phase 3 study. Lancet Oncol.

[84] Estevez LG, Gradishar WJ (2004). Evidence-based use of neoadjuvant taxane in operable and inoperable breast cancer. Clin Cancer Res.

[85] Grossman HB, Natale RB, Tangen CM (2003). Neoadjuvant chemotherapy plus cystectomy compared with cystectomy alone for locally advanced bladder cancer. N Engl J Med.

[86] Fisher B, Brown A, Mamounas E (1997). Effect of preoperative chemotherapy on local-regional disease in women with operable breast cancer: findings from National Surgical Adjuvant Breast and Bowel Project B-18. J Clin Oncol.

[87] Medical Research Council Oesophageal Cancer Working G (2002). Surgical resection with or without preoperative chemotherapy in oesophageal cancer: a randomised controlled trial. Lancet.

[88] Tarhini AA, Edington H, Butterfield LH (2014). Immune monitoring of the circulation and the tumor microenvironment in patients with regionally advanced melanoma receiving neoadjuvant ipilimumab. PLoS ONE.

[89] Tarhini AA, Zahoor H, Lin Y (2015). Baseline circulating IL-17 predicts toxicity while TGF-beta1 and IL-10 are prognostic of relapse in ipilimumab neoadjuvant therapy of melanoma. J Immunother Cancer.

[90] Salama AK, Flaherty KT (2013). BRAF in melanoma: current strategies and future directions. Clin Cancer Res.

[91] Hu-Lieskovan S, Robert L, Homet Moreno B (2014). Combining targeted therapy with immunotherapy in BRAF-mutant melanoma: promise and challenges. J Clin Oncol.

[92] Tarhini AA (2015). Neoadjuvant therapy for melanoma: a promising therapeutic approach and an ideal platform in drug development. Am Soc Clin Oncol Educ Book.

[93] Hodi FS, O’Day SJ, McDermott DF (2010). Improved survival with ipilimumab in patients with metastatic melanoma. N Engl J Med.

[94] Schadendorf D, Hodi FS, Robert C (2015). Pooled analysis of long-term survival data from Phase II and Phase III trials of ipilimumab in unresectable or metastatic melanoma. J Clin Oncol.

[95] Eggermont AM, Suciu S, Santinami M (2008). Adjuvant therapy with pegylated interferon alfa-2b versus observation alone in resected stage III melanoma: final results of EORTC 18991, a randomised phase III trial. Lancet.

[96] Eggermont AM, Spatz A, Robert C (2014). Cutaneous melanoma. Lancet.

[97] van Akkooi AC, Nowecki ZI, Voit C (2008). Sentinel node tumor burden according to the Rotterdam criteria is the most important prognostic factor for survival in melanoma patients: a multicenter study in 388 patients with positive sentinel nodes. Ann Surg.

[98] van der Ploeg AP, van Akkooi AC, Rutkowski P (2011). Prognosis in patients with sentinel node-positive melanoma is accurately defined by the combined Rotterdam tumor load and Dewar topography criteria. J Clin Oncol.

[99] Eggermont AM, Chiarion-Sileni V, Grob JJ (2015). Adjuvant ipilimumab versus placebo after complete resection of high-risk stage III melanoma (EORTC 18071): a randomised, double-blind, phase 3 trial. Lancet Oncol.

[100] Eggermont AM, Suciu S, MacKie R (2005). Post-surgery adjuvant therapy with intermediate doses of interferon alfa 2b versus observation in patients with stage IIb/III melanoma (EORTC 18952): randomised controlled trial. Lancet.

[101] Eggermont AM, Suciu S, Testori A (2012). Ulceration and stage are predictive of interferon efficacy in melanoma: results of the phase III adjuvant trials EORTC 18952 and EORTC 18991. Eur J Cancer.

[102] Eggermont AM, Suciu S, Rutkowski P (2016). Long term follow up of the EORTC 18952 trial of adjuvant therapy in resected stage IIB-III cutaneous melanoma patients comparing intermediate doses of interferon-alpha-2b (IFN) with observation: ulceration of primary is key determinant for IFN-sensitivity. Eur J Cancer.

[103] Suciu S, Ives N, Eggermont A (2014). Predictive importance of ulceration on the efficacy of adjuvant interferon-a (IFN): An individual patient data (IPD) meta-analysis of 15 randomized trials in more than 7500 melanoma patients (pts). J Clin Oncol.

[104] Robert C, Long GV, Brady B (2015). Nivolumab in previously untreated melanoma without BRAF mutation. N Engl J Med.

[105] Larkin J, Lao CD, Urba WJ (2015). Efficacy and safety of nivolumab in patients with BRAF V600 mutant and BRAF wild-type advanced melanoma: a pooled analysis of 4 clinical trials. JAMA Oncol.

[106] Robert C, Schachter J, Long GV (2015). Pembrolizumab versus ipilimumab in advanced melanoma. N Engl J Med.

[107] Weber J, Grob JJ, Margolin KA (2015). A Phase III study (CheckMate 238) of adjuvant immunotherapy with nivolumab (NIVO) versus ipilimumab (IPI) after complete resection of stage IIIb/c or stage IV melanoma (MEL) in patients (pts) at high risk for recurrence. J Transl Med.

[108] Eggermont AM, Maio M, Robert C (2015). Immune checkpoint inhibitors in melanoma provide the cornerstones for curative therapies. Semin Oncol.

[109] Zarour HM (2016). Reversing T-cell dysfunction and exhaustion in cancer. Clin Cancer Res.

[110] Fourcade J, Sun Z, Benallaoua M (2010). Upregulation of Tim-3 and PD-1 expression is associated with tumor antigen-specific CD8+ T cell dysfunction in melanoma patients. J Exp Med.

[111] Fourcade J, Sun Z, Pagliano O (2012). CD8(+) T cells specific for tumor antigens can be rendered dysfunctional by the tumor microenvironment through upregulation of the inhibitory receptors BTLA and PD-1. Cancer Res.

[112] Fourcade J, Sun Z, Pagliano O (2014). PD-1 and Tim-3 regulate the expansion of tumor antigen-specific CD8(+) T cells induced by melanoma vaccines. Cancer Res.

[113] Chauvin JM, Pagliano O, Fourcade J (2015). TIGIT and PD-1 impair tumor antigen-specific CD8(+) T cells in melanoma patients. J Clin Invest..

[114] Jensen SM, Twitty CG, Maston LD (2012). Increased frequency of suppressive regulatory T cells and T cell-mediated antigen loss results in murine melanoma recurrence. J Immunol.

[115] Yewdell JW (2011). DRiPs solidify: progress in understanding endogenous MHC class I antigen processing. Trends Immunol.

[116] Li Y, Wang LX, Yang G (2008). Efficient cross-presentation depends on autophagy in tumor cells. Cancer Res.

[117] Twitty CG, Jensen SM, Hu HM (2011). Tumor-derived autophagosome vaccine: induction of cross-protective immune responses against short-lived proteins through a p62-dependent mechanism. Clin Cancer Res.

[118] Sanborn R, Boulmay B, Li R (2015). Preliminary analysis of immune responses in patients enrolled in a Phase II trial of cyclophosphamide with allogenic dribble vaccine alone (DPV-001) or with GM-CSF or imiquimod for adjuvant treatment of stage IIIa or IIIb NSCLC. J Immunother Cancer.

[119] Page DB, Hulett TW, Hilton TL, Hu HM, Urba WJ, Fox BA (2016). Glimpse into the future: harnessing autophagy to promote anti-tumor immunity with the DRibbles vaccine. J Immunother Cancer.

[120] Lee DW, Kochenderfer JN, Stetler-Stevenson M (2015). T cells expressing CD19 chimeric antigen receptors for acute lymphoblastic leukaemia in children and young adults: a phase 1 dose-escalation trial. Lancet.

[121] Stroncek DF, Ren J, Lee DW (2016). Myeloid cells in peripheral blood mononuclear cell concentrates inhibit the expansion of chimeric antigen receptor T cells. Cytotherapy.

[122] Ludwig JA, Weinstein JN (2005). Biomarkers in cancer staging, prognosis and treatment selection. Nat Rev Cancer.

[123] Ribas A, Robert C, Hodi FS (2015). Association of response to programmed death receptor 1 (PD-1) blockade with pembrolizumab (MK-3475) with an interferon-inflammatory immune gene signature. J Clin Oncol.

[124] Seiwert TY, Burtness B, Weiss J (2015). Inflamed-phenotype gene expression signatures to predict benefit from the anti-PD-1 antibody pembrolizumab in PD-L1+ head and neck cancer patients. J Clin Oncol.

[125] Gajewski TF, Schreiber H, Fu YX (2013). Innate and adaptive immune cells in the tumor microenvironment. Nat Immunol.

[126] Spranger S, Sivan A, Corrales L (2016). Tumor and host factors controlling antitumor immunity and efficacy of cancer immunotherapy. Adv Immunol.

[127] Sivan A, Corrales L, Hubert N (2015). Commensal bifidobacterium promotes antitumor immunity and facilitates anti-PD-L1 efficacy. Science.

[128] Taube JM, Anders RA, Young GD (2012). Colocalization of inflammatory response with B7-h1 expression in human melanocytic lesions supports an adaptive resistance mechanism of immune escape. Sci Transl Med.

[129] Taube JM, Klein A, Brahmer JR (2014). Association of PD-1, PD-1 ligands, and other features of the tumor immune microenvironment with response to anti-PD-1 therapy. Clin Cancer Res.

[130] Topalian SL, Hodi FS, Brahmer JR (2012). Safety, activity, and immune correlates of anti-PD-1 antibody in cancer. N Engl J Med.

[131] Frederick DT, Piris A, Cogdill AP (2013). BRAF inhibition is associated with enhanced melanoma antigen expression and a more favorable tumor microenvironment in patients with metastatic melanoma. Clin Cancer Res.

[132] Cooper ZA, Frederick DT, Juneja VR (2013). BRAF inhibition is associated with increased clonality in tumor-infiltrating lymphocytes. Oncoimmunology.

[133] Cooper ZA, Juneja VR, Sage PT (2014). Response to BRAF inhibition in melanoma is enhanced when combined with immune checkpoint blockade. Cancer Immunol Res.

[134] Chen PL, Roh W, Reuben A (2016). Analysis of immune signatures in longitudinal tumor samples yields insight into biomarkers of response and mechanisms of resistance to immune checkpoint blockade. Cancer Discov.

[135] Filipazzi P, Valenti R, Huber V (2007). Identification of a new subset of myeloid suppressor cells in peripheral blood of melanoma patients with modulation by a granulocyte-macrophage colony-stimulation factor-based antitumor vaccine. J Clin Oncol.

[136] Filipazzi P, Burdek M, Villa A (2012). Recent advances on the role of tumor exosomes in immunosuppression and disease progression. Semin Cancer Biol.

[137] Ridder K, Sevko A, Heide J (2015). Extracellular vesicle-mediated transfer of functional RNA in the tumor microenvironment. Oncoimmunology.

[138] Jacquelot N, Roberti MP, Enot DP (2016). Immunophenotyping of stage III melanoma reveals parameters associated with patient prognosis. J Invest Dermatol.

[139] Jacquelot N, Enot DP, Flament C (2016). Chemokine receptor patterns in lymphocytes mirror metastatic spreading in melanoma. J Clin Invest.

[140] Vetizou M, Pitt JM, Daillere R (2015). Anticancer immunotherapy by CTLA-4 blockade relies on the gut microbiota. Science.

[141] Lee JW, Devanarayan V, Barrett YC (2006). Fit-for-purpose method development and validation for successful biomarker measurement. Pharm Res.

[142] Dako. PD-L1 IHC 22C3 pharmDx specification sheet. 2015. http://www.dako.com/download.pdf?objectid=128206001. Accessed 3 Oct 2016.

[143] Cree IA, Deans Z, Ligtenberg MJ (2014). Guidance for laboratories performing molecular pathology for cancer patients. J Clin Pathol.

[144] Pant S, Weiner R, Marton MJ (2014). Navigating the rapids: the development of regulated next-generation sequencing-based clinical trial assays and companion diagnostics. Front Oncol.

[145] Rehm HL, Bale SJ, Bayrak-Toydemir P (2013). ACMG clinical laboratory standards for next-generation sequencing. Genet Med.

[146] Landis JR, Koch GG (1977). The measurement of observer agreement for categorical data. Biometrics.

[147] Mandrekar SJ, Sargent DJ (2009). Clinical trial designs for predictive biomarker validation: theoretical considerations and practical challenges. J Clin Oncol.

